# Integrating network pharmacology and experimental validation strategies to investigate the mechanisms and key flavonoids in medicinal and edible citrus plants against Alzheimer’s disease

**DOI:** 10.3389/fnagi.2026.1801263

**Published:** 2026-04-28

**Authors:** Xin-Yi Liu, Yi-Zhi Yan, An-Jun Jiang, Si-Jie Tan, Ying-Yan Fang, Peng Zeng

**Affiliations:** 1 Department of Histology and Embryology, School of Basic Medicine, Hengyang Medical School, University of South China, Hengyang, China; 2 Hubei Key Laboratory for Kidney Disease Pathogenesis and Intervention, Hubei Polytechnic University School of Medicine, Huangshi, China

**Keywords:** Alzheimer’s disease, BV2 microglial cells, citrus plants, ferroptosis, flavonoids, pathophysiological processes

## Abstract

**Introduction:**

Due to the complexity of the Alzheimer’s disease (AD) pathophysiological processes, there is currently a lack of effective therapeutic drugs. The medicinal and edible substances have multiple advantages in treating AD, but their specific components and mechanisms remain unclear. This study aims to investigate the potential mechanisms of flavonoids in medicinal and edible citrus plants in treating AD and their key phytochemicals.

**Methods:**

We collected flavonoids identified by UHPLC-Q-TOF-MS/MS in citrus plants from the literatures and evaluate their pharmacological and toxicological parameters. We obtained and systematically analyzed the action targets of the flavonoids of citrus plants and screened the targets related to AD key pathophysiological processes and the corresponding phytochemicals. The results of network pharmacological analysis were further validated through molecular docking, GEO database, and BV2 microglial cells.

**Results:**

A total of 51 flavonoids in medicinal and edible citrus plants were identified, which exhibit favorable pharmacological properties and safety profiles. Multiple flavonoid compounds such as isoquercitrin, astragalin, cynaroside, troxerutin and lonicerin serve as potential acetylcholinesterase inhibitors for the symptomatic treatment of AD. The study identified 45 flavonoids in citrus plants that correspond to 304 AD-related targets, which are involved in multiple pathophysiological processes. Quercetin, nobiletin, hesperidin, apigenin, HTMF, tangeretin and hesperetin have been identified as the key flavonoids of citrus plants that regulate the pathogenesis of AD in a multitargeted manner. The flavonoids of citrus plants primarily regulate the core targets AKT1, TNF, IL6, TP53, IL1B, STAT3, INS, JUN, CASP3 and CTNNB1. Targeting ferroptosis is one of the mechanisms by which citrus plants to ameliorate AD. *In vitro* experiments also demonstrated that hesperidin and naringin alleviated LPS-induced pro-inflammatory activation of BV2 cells.

**Conclusion:**

The various citrus plants flavonoids examined in this study exhibit significant potential for clinical translation, particularly in the early prevention and adjuvant treatment of AD.

## 1 Introduction

Alzheimer’s disease (AD) is a neurodegenerative disorder characterized by an insidious onset and progressive deterioration. Its clinical manifestations include progressive memory loss, language impairment, cognitive dysfunction, and motor disturbances (Zheng and Wang, 2025). In 2019, the global number of dementia patients was approximately 57.4 million, with AD accounting for 60%–80% of dementia cases. Without effective prevention and treatment measures, the number of dementia patients is projected to rise to 152 million by 2050, with AD being the most prevalent cause of dementia in individuals over the age of 65 (Alzheimer’s Association, 2023). According to the China Alzheimer report 2024, there are currently nearly 17 million dementia patients in China, accounting for nearly 30% of the global total. Given the irreversible nature of AD and low patient compliance, the increasing social burden it imposes, and the limitations of existing treatments, there is an urgent need for the development of therapeutic drugs for AD.

Although several hypotheses regarding the pathogenesis of AD have been proposed, the exact mechanisms underlying AD remain unclear, particularly due to the lack of understanding of the pathological mechanisms during the preclinical stage of AD (without cognitive impairment). Currently, the pathological mechanisms of AD involve multiple processes, including β-amyloid (Aβ) deposition, tau protein hyperphosphorylation, neuroinflammation, oxidative stress, synaptic damage, and neuronal death (Long et al., 2022a,b; Wang et al., 2025; Zhang et al., 2025; Zheng and Wang, 2025). The decline in acetylcholine (ACh) levels synthesized and released by cholinergic neurons in the basal forebrain is one of the core mechanisms underlying cognitive dysfunction in AD patients. Currently, FDA-approved clinical drugs for AD primarily alleviate symptoms by indirectly increasing the concentration of ACh in the brain through the inhibition of ACh degradation. However, merely increasing ACh levels does not reverse the progression of AD or prevent the ongoing damage to cholinergic neurons. Aβ-targeting drugs such as aducanumab and lecanemab, while capable of clearing Aβ plaques, only slightly delay cognitive decline and may cause severe side effects such as cerebral edema and cerebral hemorrhage (Terao and Kodama, 2025). More critically, they do not significantly improve tau protein pathology and neuronal loss, and cannot halt disease progression. The long-term use of medications for treating AD also exhibits significant side effects. Due to the complexity of AD pathology, future treatments must incorporate multi-target strategies, including synergistic interventions at various stages, to manage AD more effectively. Certain medicinal and edible substances exhibit a range of biological activities against AD, with high safety profiles, highlighting their unique advantages and potential in the development of anti-AD pharmaceuticals.

The medicinal and edible substances refer to natural materials that can be consumed as part of daily diet while also possessing medicinal efficacy. The core advantages of medicinal and edible substances in the treatment of AD are reflected in three aspects. First, medicinal and edible substances have a solid safety foundation, making them suitable for long-term intervention. Second, the synergistic effects of multiple components match the complex pathology of AD. Third, they are widely sourced and cost-controllable. Recent studies have revealed that the active ingredients in various medicinal and edible substances can ameliorate the pathological features and cognitive impairments associated with AD through multiple mechanisms (El-Maraghy et al., 2023; Zhu et al., 2024). Citrus, belonging to the genus Citrus in the family Rutaceae, represents a significant economic crop in China (Dhuique-Mayer and Servent, 2025). Its fruits are rich in bioactive phytochemicals such as flavonoids, coumarins, and alkaloids, and possess various biological activities including anti-oxidant (Borghi and Pavanelli, 2023; Wang Y. et al., 2022), anti-inflammatory (Fontana et al., 2022), anti-bacterial (Ciriminna et al., 2025), and anti-cancer (Cirmi et al., 2018; Hermawan et al., 2025) properties. In Asian countries such as China and South Korea, citrus plants are extensively utilized as medicinal herbs in traditional medicine, owing to their significant medicinal value (Maksoud et al., 2021). Daily consumption of citrus plants can also alleviate or treat various health issues, including cough, phlegm, indigestion, and inflammation (Lu et al., 2023). In addition to the overall observation of macroscopic therapeutic effects, a precise analysis of the pharmacodynamic components at the compound level is essential for the medicinal and edible substances.

This study focuses on 6 common medicinal and edible substances in citrus plants, including *Fructus Aurantii* (枳壳), *Fructus Aurantii Immaturus* (枳实), *Citri Sarcodactylis Fructus* (佛手), Zhique (the dried fruit of *Citrus grandis × junos*) (枳雀), *Exocarpium Citri Grandis* (化橘红), and *Citrus Reticulata Blanco* (广陈皮). The medicinal and edible citrus plants contain primary metabolites, including soluble sugars, dietary fiber, organic acids, and vitamins, all of which are essential nutrients for human health. However, its biological activities primarily derives from its unique active components, which include secondary metabolites such as flavonoids, coumarins, alkaloids, limonoids, and volatile oils (Farag et al., 2020). Flavonoids constitute the most abundant class of secondary metabolites found in both medicinal and edible citrus plants (Xu et al., 2025). Recent studies have demonstrated that flavonoids significantly contribute to the medicinal value of medicinal and edible citrus plants, exhibiting high safety profiles and a variety of biological activities (Jeffries et al., 2025; Jomova et al., 2025). The dietary intake of flavonoid-rich food sources significantly enhances intelligence and delays or prevents aging as well as neurodegenerative diseases, including AD (Calderaro et al., 2022; Minocha et al., 2022). The pathogenesis of AD involves multiple signaling pathways, and treatments targeting a single pathway may alleviate symptoms but cannot influence the disease progression. This study focuses on the potential mechanisms of flavonoids in medicinal and edible citrus plants in treating AD and their key phytochemicals. This study not only facilitates the global dissemination and application of traditional medical concepts but also provides a research direction characterized by Chinese elements for international studies on the prevention and treatment of AD.

## 2 Materials and methods

### 2.1 Drugs and antibodies

LPS (≥500,000 EU/mg, # BS904-10 mg) was from Biosharp (Beijing, China). Hesperidin (purity = 98.14%, CAS #: 520-26-3) and naringin (purity = 99.65%, CAS #: 10236-47-2) were procured from MedChemExpress (Shanghai, China) and dissolved in DMSO. The primary antibodies specific for β-actin (# 66009-1-lg), cyclooxygenase 2 (Cox2, # 27308-1-AP), IL1β (# 26048-1-AP), TNF-α (# 29652-1-AP), NF-κB p65 (# 80979-1-RR), p-NF-κB p65 (Ser468,# 82,335-1-RR), JUN (# 24909-1-AP) and p-JUN (Ser73, # 28891-1-AP) were purchased from Proteintech (Wuhan, China). JNK (# AF6318) and p-JNK (Thr183 + Tyr185, # AF3318) were purchased from Affinity Biosciences (Jiangsu, China). Horseradish peroxidase (HRP)-labeled goat anti-rabbit/mouse (# A21020/A21010) IgG was used as secondary antibody and purchased from Abbkine (Wuhan, China).

### 2.2 Collect the main flavonoids in medicinal and edible citrus plants and evaluation of their pharmacological parameters

This study focuses on flavonoids, the most abundant secondary metabolites in medicinal and edible citrus plants. By conducting a thorough search of the PubMed database, we compiled a list of 51 flavonoids identified in previous studies utilizing the UHPLC-Q-TOF-MS/MS method (Fu et al., 2020; Guo et al., 2021; He et al., 2018; Li et al., 2014; Liu et al., 2017; Qiao et al., 2021; Wang K. et al., 2021; Xie et al., 2021; Yan et al., 2021; Zhang et al., 2020; Zhao et al., 2015; Zheng et al., 2019; Zhu et al., 2020). The SMILES of each flavonoid were downloaded from the PubChem database. Next, we input the SMILES of flavonoids into the SwissADME tool^1^ to calculate the Lipinski’s rule of five (RO5) of each flavonoid. Compounds that comply with RO5 indicate better oral bioavailability.

### 2.3 Analysis of toxicological parameters of main flavonoids in citrus plants

The safety and efficacy of compounds are the determining factors for the success of drug development. This study calculated the toxicological parameters of flavonoids using the ProToxII^2^ (Banerjee et al., 2018). In toxicological parameters, oral toxicity includes LD50 and toxicity class, organ toxicity covers hepatotoxicity, and toxicity endpoints include cytotoxicity, mutagenicity, immunotoxicity and carcinogenicity. Referring to previous studies (Deng et al., 2025; Yan et al., 2025), compounds classified with an acute toxicity class greater than Class 3 and exhibiting fewer than three positive toxicity endpoints were deemed non-toxic.

### 2.4 Screening of potential targets of main flavonoids in citrus plants

This study employed three strategies, reference mining, database mining and structure-based prediction to identify potential targets of flavonoids in citrus plants. Screening strategies reference mining and database mining are implemented based on the “Related targets” function of the HERB database^3^ (Fang et al., 2021). For flavonoids that could not be retrieved in the HERB database, we employed the SwissTargetPrediction database to predict potential targets based on their chemical structures (Daina et al., 2019). We ranked the confidence levels of the potential targets of flavonoids as reference mining being greater than database mining, which in turn is greater than structure-based prediction. We further utilized the PantherDB database^4^ to classify the protein functions of citrus plants targets.

### 2.5 Analysis of acetylcholinesterase (AChE) levels in different brain regions of AD patients

The primary function of the enzyme AChE within the cholinergic system is to efficiently hydrolyze the neurotransmitter ACh into choline and acetic acid. The decline in cognitive function in AD patients is closely associated with changes in the levels and activity of AChE in specific brain regions. We retrieved the expression profiles of AChE or other anti-AD targets in different brain regions of AD patients and the control group using the Differential Expression module of the AlzData database^5^ (Xu et al., 2018). The receiver operating characteristic (ROC) curve was plotted using an online tool.^6^

### 2.6 Molecular docking simulations and molecular dynamics (MD) simulations

The automated molecular docking simulations were carried out using the molecular docking procedure known as LeDock^7^ (Wang et al., 2016). In summary, three-dimensional structures of the target proteins were obtained from the RCSB Protein Data Bank (PDB database, http://www.rcsb.org/) (Burley et al., 2022), while three-dimensional files in SDF format for flavonoids were sourced from the PubChem database. All the prepared ligand files were converted into mol2 format using Open Babel software. The protein crystal structures of human for AChE (PDB ID: 4BDT), BChE (PDB ID: 6I2T), GSK3β (PDB ID: 2O5K), IL1β (PDB ID: 5R85), NOS1 (PDB ID: 5UO1), NOS2 (PDB ID: 3HR4), PTGS2 (PDB ID: 5F19), and TNF-α (PDB ID: 2AZ5) were downloaded in PDB format. To prepare the receptor files, the LePro tool facilitated processes such as hydrogenation and binding pocket identification. LePro tool sets the root mean square deviation (RMSD) to 1.0 Å with a default binding pose number of 20, and the binding pocket is set as a rectangular box. The binding pockets of each protein are listed in Table 1. The scoring function, set to default parameters, was employed to calculate the docking score (in kcal/mol). Additionally, the interaction residues and hydrogen bond lengths between the receptor and ligand were visualized using LigPlot^8^ (Laskowski and Swindells, 2011).

**Table 1:** Binding pockets information of receptor proteins.

Receptors	x_min_ (Å)	x_max_ (Å)	y_min_ (Å)	y_max_ (Å)	z_min_ (Å)	z_max_ (Å)
AChE	−8.72	4.1	−44	−28.14	−58.85	−42.83
BChE	149.81	164.61	185.69	196.06	130.01	144.16
GSK3β	−1.43	18.04	18.44	38.85	−20.29	−2.07
IL1β	33.68	44.71	−4.4	10.12	67.17	79.34
NOS1	111.62	129.97	238.82	258.99	351.3	364.82
NOS2	−5.93	7.95	0.26	18.11	−74.61	−54.76
PTGS2	17.79	37.92	21.81	39.07	53.96	72.38
TNF-α	−27.7	−10.63	66.34	82.57	25.6	42.08

The docking conformation with the lowest score, considered optimal, was selected for subsequent MD simulation analysis. These MD simulations were conducted using the GROMACS 2024.1 software package. Ligand parameters were derived from the General Amber Force Field (GAFF2), with charges calculated using the AM1-BCC method. The AMBER ff14sb_OL15 protein force field was employed in conjunction with the TIP3P water model. Prior to MD simulation, energy minimization was performed to eliminate steric clashes and local energy minima in the system. Production MD simulations were carried out at a temperature of 300 K for a duration of 200 ns, with a time step of 2 fs, under conditions of constant volume and temperature. To evaluate the stability of the complex structure, the RMSD was analyzed. For more detailed steps, please refer to our previously published paper (Yan et al., 2025).

### 2.7 Protein-protein interaction (PPI) network construction and core targets screening

This study integrated the STRING 12.0^9^ and Cytoscape 3.10.3 for PPI network visualization and analysis (Szklarczyk et al., 2025). When constructing the PPI, we selected the species as *homo sapiens* and the confidence level as medium (combined_score ≥ 0.4). This study used the Network Analysis plugin in Cytoscape 3.10.3 to calculate the degree value (the number of edges owned by a node) of each node in the PPI network. The higher the degree of a node, the greater its importance in network regulation. Among the PPI network, the top 20 nodes ranked by degree are identified as core targets.

### 2.8 Screening of anti-AD targets in citrus plants and their corresponding flavonoids

We comprehensively screened potential anti-AD targets in citrus plants, achieving a paradigm shift from intersection analysis to full-spectrum analysis. We employed three methods to comprehensively screen anti-AD targets and corresponding flavonoids in citrus plants (Zeng et al., 2022). First, based on the KEGG database, we identified the citrus plant targets involved in the Alzheimer disease (hsa05010) pathway, which primarily involves Aβ generation and deposition, tau hyperphosphorylation and neurofibrillary tangles, neuroinflammation, and oxidative stress. Second, we conducted a disease ontology (DO) enrichment analysis on the targets of citrus plants, with a focus on screening flavonoid targets enriched in Alzheimer’s disease (DOID:10652). The interaction between Aβ and tau pathology constitutes a vicious cycle in the progression of AD. Thirdly, based on the AlzData database, we analyzed the targets among all citrus plant potential targets that are significantly associated with Aβ pathology and tau pathology. Through the “Single Cell Expression” module of the AlzData database, we analyzed the cellular localization of citrus plant anti-AD targets located in the human brain, including neurons, astrocytes, microglia, endothelial cells and oligodendrocytes.

### 2.9 Gene set enrichment analysis (GSEA)

The GSE5281 dataset (Liang et al., 2007) was retrieved from the GEO database using the GEOquery R package. It comprises 23 samples from the human hippocampus, including 13 control samples (age: 79.6 ± 2.6 years) and 10 AD samples (age: 77.8 ± 1.8 years). Hippocampus is the main brain region affected in the early stage of AD. To identify differentially expressed genes (DEGs) in the AD hippocampus, limma package in R version 4.2.1 was utilized with cut-offs of adjusted *p* value < 0.05 and fold change > 2. The GSEA was performed using ClusterProfiler R package (version 4.2.1). For each GSEA, the normalized enrichment score (NES) and *p* value were specified. Pathway with *p* value < 0.05 was considered as significantly enriched pathway.

### 2.10 Gene ontology (GO) enrichment analysis

ClusterProfiler R package was used for GO enrichment analysis. The *p* values were adjusted using a Benjamini-Hochberg approach. Statistical significance was denoted if adjust *p* value <0.05. The GO analysis encompassed biological process, molecular function, and cellular component.

### 2.11 Cell culture and cell counting kit-8 (CCK8) assay

The immortalized BV2 microglial cell line was obtained from Boster Biological Technology (product number: XBS0421-0017, Wuhan, China). Cells were cultured in *α*-MEM supplemented with 10% FBS and penicillin/streptomycin at 37 °C a humidified incubator under a 95%/5% (v/v) mixture of air and CO_2_. We established an *in vitro* cell model of neuroinflammation by treating BV2 cells with LPS (1 μg/mL) for 24 h (Guo et al., 2019; Wang et al., 2024).

For CCK-8 assay, BV2 cells were seeded in 96-well plates at a appropriate density. Various concentrations of hesperidin (0, 10, 20, 30, 40, 50 μM) were used to treat cells for 24 h. After 24 h exposure, the medium was replaced with 100 μL of fresh medium containing 10% CCK-8 reagent (# C0038, Beyotime, Shanghai, China) for 60 min at 37 °C. Optical densities of CCK8 were measured using a Multiskan FC microplate reader (Thermo Scientific, USA) at a wavelength of 450 nm.

### 2.12 Microglia and neurons co-culture system

HT22 mouse hippocampal neuronal cell line was obtained from iCell Bioscience Inc. (Shanghai, China). Cells were cultured in a cell culture flask containing DMEM with 10% FBS in a humidified incubator under a 95%/5% (v/v) mixture of air and CO_2_ (Chang et al., 2023). Regarding the microglial cells BV2 and HT22 co-culture system, BV2 cells were seeded in transwell (0.4 μm pores) upper chamber and the HT22 cells were cultured in the plates. The co-culture system was harvested 24 h after the specified intervention.

### 2.13 Western blotting

After treating BV2 cells with 1 ug/ml of LPS or flavonoids for 24 h, the cellular proteins were extracted. Protein concentrations were determined by a bicinchoninic acid kit (Beyotime, Shanghai, China). Western blotting analysis was performed as described in detail previously (Zeng et al., 2022; Zhu et al., 2025). Each antibody was dissolved in SuperKine™ Enhanced Antibody Dilution Buffer (# BMU103-CN, Abbkine, Wuhan, China). The PVDF membranes were incubated with primary antibodies at 4 °C overnight. Signals were visualized with an enhanced chemiluminescence imaging system (SCG-W2000, Servicebio, Wuhan, China). Quantitative analysis for western blotting was performed with ImageJ software (NIH, Bethesda, MD, USA).

### 2.14 Statistical analysis

All data were expressed as the means ± standard error of mean (SEM). Two-group comparisons were statistically evaluated by two-tailed unpaired *t*-test, and one-way ANOVA analysis followed by an LSD multiple comparison test for three-group comparisons. SPSS 19.0 statistical software (SPSS, Chicago, IL, USA) was used for statistical analysis. Data visualization was performed using Prism 8.0 (GraphPad Software, USA). The significance level was defined as *p* < 0.05.

## 3 Results

### 3.1 Flavonoids in medicinal and edible citrus plants and their safety evaluation

**Figure 1:**
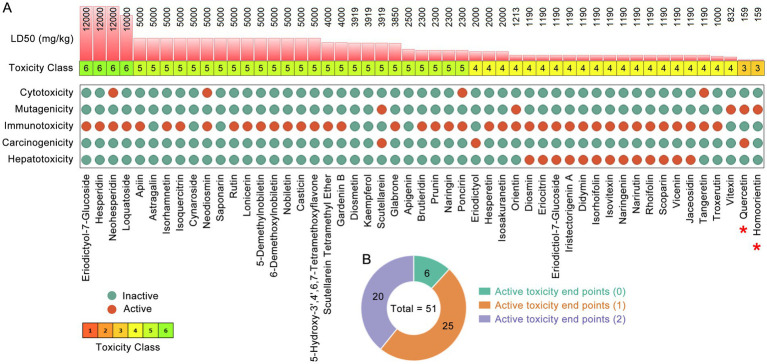
Flavonoids in medicinal and edible citrus plants and their safety assessment. **(A)** Toxicological parameters of 51 flavonoids in medicinal and edible citrus plants. The red and green circles represent active and inactive toxicology end point, respectively. **(B)** The donut chart represents the number of active toxicological end point for flavonoids in citrus plants.

Flavonoids are the most abundant class of secondary metabolites in medicinal and edible citrus plants. Based on the previous UHPLC-Q-TOF-MS/MS data, a total of 51 flavonoids in medicinal and edible citrus plants were identified (Table 2). The distribution of 51 flavonoids in 6 medicinal and edible citrus plants is shown in Supplementary Figure S1. In Table 2, the 21 flavonoids labeled by hashtag (#) fully comply with RO5, indicating that these phytochemicals possess favorable pharmacokinetic properties. We used the ProTox-II web server (Banerjee et al., 2018) to evaluate the toxicological parameters of flavonoids in citrus plants in silico. According to the criterion that acute toxicity greater than level 3 and fewer than 3 positive toxicity endpoints indicate non-toxicity (Wei et al., 2021; Yan et al., 2025), all flavonoids in citrus plants except quercetin and homoorientin were identified as non-toxic (Figure 1A). Among these flavonoids, eriodictyol-7-glucoside, hesperidin and neohesperidin exhibit the highest safety, with an LD50 of 12,000 mg/kg, followed by loquatoside (LD50 = 10,000 mg/kg). Among the flavonoids in citrus plants, there are 6, 25, and 20 phytochemicals with 0, 1, and 2 active toxicity endpoints, respectively (Figure 1B). These results indicate that the flavonoids present in medicinal and edible citrus plants demonstrate favorable pharmacological properties and safety profiles.

**Table 2:** Flavonoids in medicinal and edible citrus plants.

Flavonoids	Formula	MW (g/mol)	Hdon	Hacc	Rbon	LogP	TPSA (Å)
5-Demethylnobiletin #	C_20_H_20_O_8_	388.37	1	8	6	2.78	96.59
6-Demethoxylnobiletin #	C_20_H_20_O_7_	372.37	0	7	6	2.98	76.36
Apigenin #	C_15_H_10_O_5_	270.24	3	5	1	2.11	90.9
Apiin	C_26_H_28_O_14_	564.49	8	14	7	−0.68	228.97
Astragalin	C_21_H_20_O_11_	448.38	7	11	4	−0.09	190.28
Brutieridin	C_34_H_42_O_19_	754.69	9	19	13	−0.85	297.89
Casticin #	C_19_H_18_O_8_	374.34	2	8	5	2.51	107.59
Cynaroside	C_21_H_20_O_11_	448.38	7	11	4	0.15	190.28
Didymin	C_28_H_34_O_14_	594.56	7	14	7	−0.57	214.06
Diosmetin #	C_16_H_12_O_6_	300.26	3	6	2	2.19	100.13
Diosmin	C_28_H_32_O_15_	608.54	8	15	7	−0.52	238.2
Eriocitrin	C_27_H_32_O_15_	596.53	9	15	6	−1.28	245.29
Eriodictiol-7-Glucoside	C_22_H_24_O_11_	464.42	6	11	5	0.34	175.37
Eriodictyol #	C_15_H_12_O_6_	288.25	4	6	1	1.45	107.22
Eriodictyol-7-Glucoside	C_21_H_22_O_11_	450.39	7	11	4	−0.32	186.37
Gardenin B #	C_19_H_18_O_7_	358.34	1	7	5	2.82	87.36
Glabrone #	C_20_H_16_O_5_	336.34	2	5	1	3.13	79.9
Hesperetin #	C_16_H_14_O_6_	302.28	3	6	2	1.91	96.22
Hesperidin	C_28_H_34_O_15_	610.56	8	15	7	−1.06	234.29
Homoorientin	C_21_H_20_O_11_	448.38	8	11	3	−0.34	201.28
HTMF #	C_19_H_18_O_7_	358.34	1	7	5	2.89	87.36
Iristectorigenin A #	C_17_H_14_O_7_	330.29	3	7	3	2.09	109.36
Isoquercitrin	C_21_H_20_O_12_	464.38	8	12	4	−0.48	210.51
Isorhamnetin #	C_16_H_12_O_7_	316.26	4	7	2	1.65	120.36
Isorhoifolin	C_27_H_30_O_14_	578.52	8	14	6	−0.64	228.97
Isosakuranetin #	C_16_H_14_O_5_	286.28	2	5	2	2.25	75.99
Isovitexin	C_21_H_20_O_10_	432.38	7	10	3	−0.02	181.05
Jaceosidin #	C_17_H_14_O_7_	330.29	3	7	3	1.7	105
Kaempferol #	C_15_H_10_O_6_	286.24	4	6	1	1.58	111.13
Lonicerin	C_27_H_30_O_15_	594.52	9	15	6	−0.95	249.2
Loquatoside	C_20_H_22_O_11_	438.38	8	11	3	−0.91	189.53
Naringenin #	C_15_H_12_O_5_	272.25	3	5	1	1.84	86.99
Naringin	C_27_H_32_O_14_	580.53	8	14	6	−0.87	225.06
Narirutin	C_27_H_32_O_14_	580.53	8	14	6	−1.06	225.06
Neodiosmin	C_28_H_32_O_15_	608.54	8	15	7	−0.41	238.2
Neohesperidin	C_28_H_34_O_15_	610.56	8	15	7	−1.02	234.29
Nobiletin #	C_21_H_22_O_8_	402.39	0	8	7	3.02	85.59
Orientin	C_21_H_20_O_11_	448.38	8	11	3	−0.47	201.28
Poncirin	C_28_H_34_O_14_	594.56	7	14	7	−0.52	214.06
Prunin	C_21_H_22_O_10_	434.39	6	10	4	0.23	166.14
Quercetin #	C_15_H_10_O_7_	302.24	5	7	1	1.23	131.36
Rhoifolin	C_27_H_30_O_14_	578.5	8	14	6	−0.2	225
Rutin	C_27_H_30_O_16_	610.5	10	16	6	−1.3	266
Saponarin	C_27_H_30_O_15_	594.52	10	15	6	−1.69	260.2
Scoparin	C_22_H_22_O_11_	462.4	7	11	4	0.2	186
Scutellarein #	C_15_H_10_O_6_	286.24	4	6	1	1.81	111.13
Scutellarein Tetramethyl Ether #	C_19_H_18_O_6_	342.34	0	6	5	3.01	67.13
Tangeretin #	C_20_H_20_O_7_	372.37	0	7	6	3.02	76.36
Troxerutin	C_33_H_42_O_19_	742.68	10	19	15	−1.47	297.12
Vicenin	C_27_H_30_O_15_	594.52	11	15	5	−2.07	271.2
Vitexin	C_21_H_20_O_10_	432.38	7	10	3	−0.02	181.05

The hashtag (#) indicates flavonoids that fully comply with the RO5. HTMF, 5-Hydroxy-3′, 4′, 6,7-Tetramethoxyflavone.

### 3.2 Flavonoids in medicinal and edible citrus plants alleviate AD symptoms by targeting AChE

**Figure 2:**
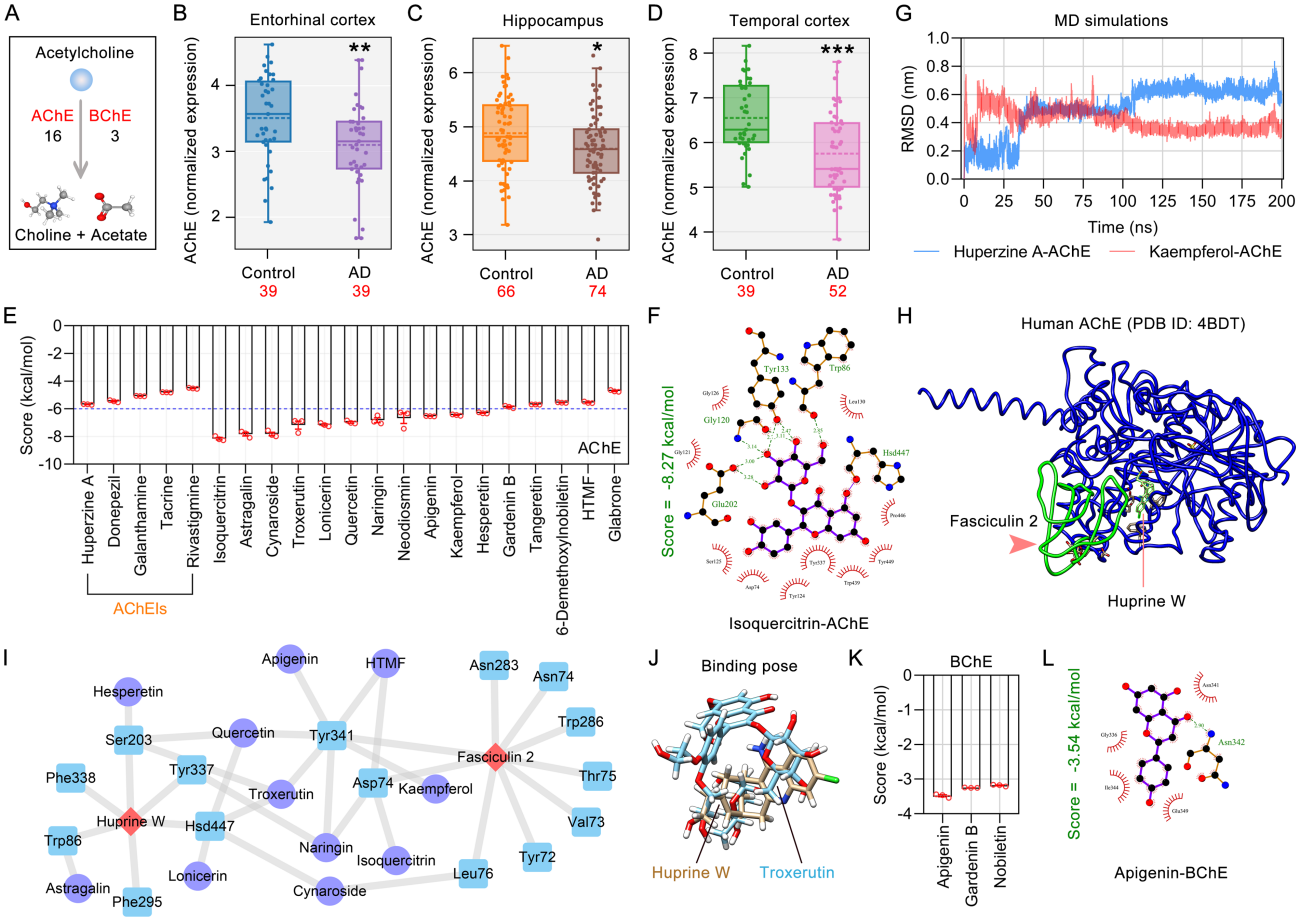
Flavonoids in medicinal and edible citrus plants have been identified as modulators of AChE. **(A)** Schematic diagram of the degradation pathway of acetylcholine in the synaptic cleft. The levels of AChE in the brains of AD patients are significantly reduced in brain regions entorhinal cortex **(B)**, hippocampus **(C)**, and temporal cortex **(D)**. **(E)** The bar chart displays the molecular docking scores between phytochemicals in citrus plants and AChE (PDB ID: 4BDT) (*n* = 3). **(F)** LigPlus schematic 2D representation of isoquercitrin-AChE interactions. The amino acid residues of AChE that interact with isoquercitrin are depicted as brown sticks and labeled in green. **(G)** RMSD profiles of the huperzine A and kaempferol after binding to AChE over 200 ns. **(H)** Crystal structure of human AChE in complex with huprine W and fasciculin 2. **(I)** The network diagram shows the AChE amino acid residues co-interacted by citrus plant flavonoids and the positive control (huprine W and fasciculin 2). **(J)** The optimal binding pose diagram of troxerutin and huprine W docked with AChE. **(K)** The bar chart illustrates the molecular docking scores between the phytochemicals in citrus plants and BChE (PDB ID: 6I2T). **(L)** LigPlus schematic 2D representation of apigenin-BChE interactions. Data were expressed as the means ± SEM. *p < 0.05, **p < 0.01, ***p < 0.001.

Current AD treatment is symptomatic therapy primarily achieved by restoring ACh levels. The synaptic cleft ACh activates the cholinergic nervous system in the brain by binding to its receptors. AChE and BChE are the enzymes responsible for the hydrolysis of ACh in the synaptic cleft. AChE is the primary enzyme, accounting for approximately 90% of ACh hydrolysis, while BChE functions as a compensatory enzyme (Figure 2A). Therefore, we selected ACHE (PDB ID: 4BDT) and BCHE (PDB ID: 6I2T) for molecular docking validation with citrus plant flavonoids.

In AlzData database, the level of AChE in AD patients was significantly increased than those in normal controls. In AlzData database, the AChE levels in entorhinal cortex, hippocampus, and temporal cortex of AD patients were significantly lower compared to the normal controls (Figures 2B–D). Sixteen flavonoids in medicinal and edible citrus plants were shown to target to target AChE, and 3 flavonoids have been proven to target BChE. Through molecular docking, we assessed the AChE-binding affinities of five clinically utilized AChE inhibitors approved by the American Food and Drug Administration (FDA), as well as flavonoids found in medicinal and edible citrus plants. The binding affinities of 12 flavonoids, including isoquercitrin, astragalin, cynaroside, troxerutin, lonicerin and quercetin, were all higher than those of all five clinically used AChE inhibitors (Figure 2E). Among them, isoquercitrin had best docking posture with energy −8.27 kcal/mol. Isoquercitrin forms 7 hydrogen bonds with amino acid residues Glu202, Gly120, Trp86 and Tyr133 in the AChE binding pocket (Figure 2F). We conducted MD simulations with a duration of 200 ns using GROMACS 2024.1 to further investigate the stability of the binding model of the ligand-protein complexes. The kaempferol-AChE complex remained stable after 9 ns of simulation, with a RMSD ranging from 0.3 to 0.5 nm (average value: 0.42 nm). This consistent RMSD range suggests that the binding mode of the ligand to the protein remained largely unchanged during the simulation period. The huperzine A-AChE complexshowed stable RMSD between 35 ns to 105 ns at RMSD about 0.5 nm, after 105 ns RMSD increases and constant at 0.66 nm. Compared with huperzine A, which has the optimal docking score with AChE among the five FDA-approved AChE inhibitors, the kaempferol-AChE complex achieves stability earlier and exhibits better structural stability of the system (Figure 2G). The PDB file for human AChE (4BDT) contains two distinct site inhibitors, Huprine W and Fasciculin 2, both of which are recognized as potent inhibitors of ACHE in research settings (Waqar and Batool, 2015) (Figure 2H). The molecular docking score of huprine W with AChE was −7.35 kcal/mol, while isoquercitrin, astragalin and cynaroside showed better docking scores with AChE than huprine W. As shown in Figure 2I, citrus plants flavonoids exhibit conserved hydrogen bonding interactions with key residues (e.g., Asp74, Hsd447, Ser203, Trp86, Tyr341) as observed for the positive control Huprine W and Fasciculin 2. Troxerutin and the AChE positive control Huprine W exhibit similar binding poses within the active pocket (Figure 2J). These findings substantiate the superior binding affinity and highlight the potential of citrus plants flavonoids as novel AChE inhibitors. In AD, the level of BChE remains stable or increases to 165% of the normal level (Mushtaq et al., 2014). For BChE, the molecular docking scores of apigenin, nobiletin and gardenin B with it are all greater than −4 kcal/mol (Figure 2K). Apigenin potentially interacts with BChE by forming a 2.90 Å hydrogen bond with the Asn342 residue (Figure 2L).

### 3.3 Targets and key flavonoids of medicinal and edible citrus plants in the treatment of AD

**Figure 3:**
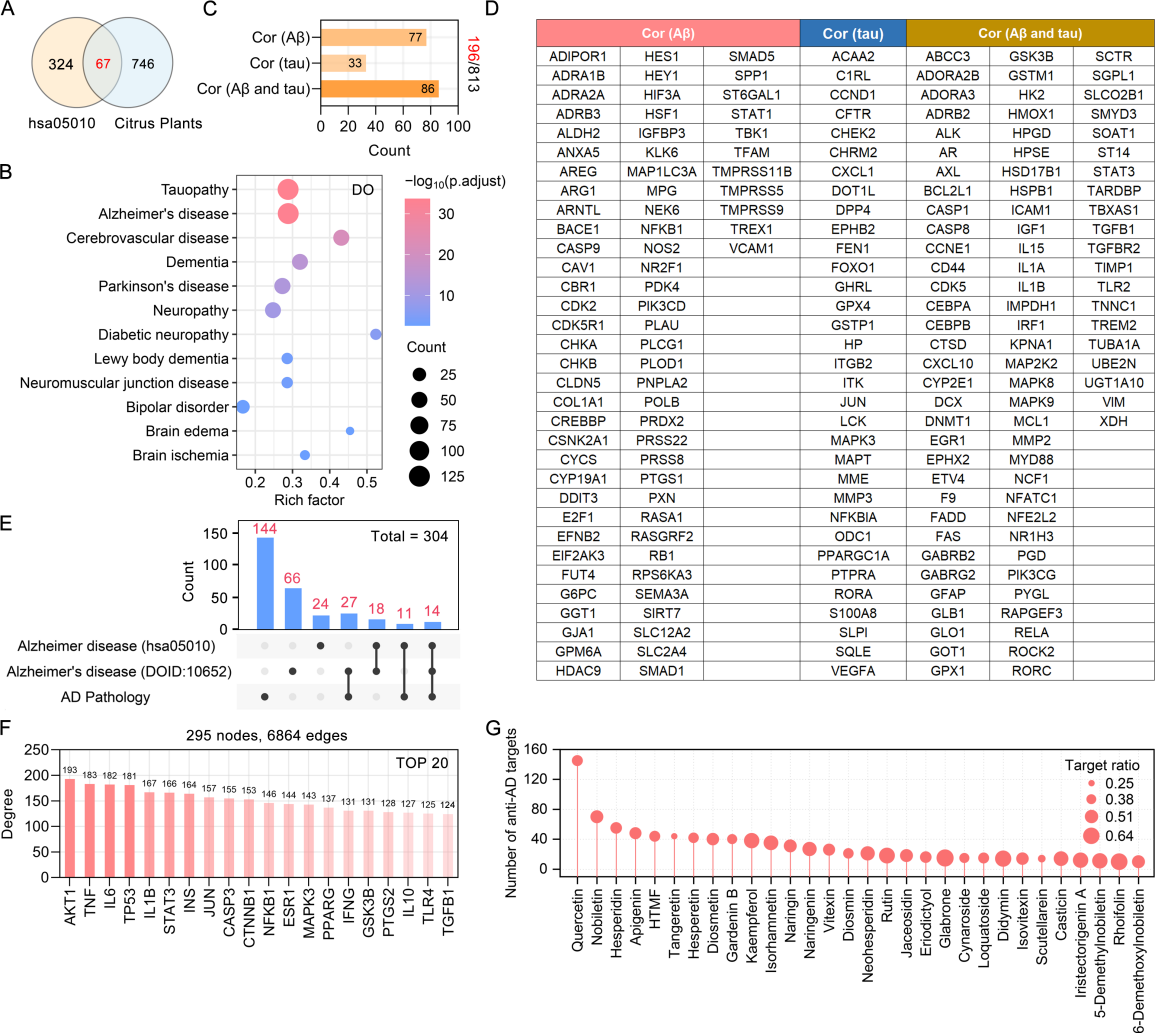
Identification of anti-AD targets and key flavonoids in medicinal and edible citrus plants. **(A)** The common targets between the potential targets of flavonoids in citrus plants and the Alzheimer disease pathway (hsa05010) targets. **(B)** Bubble chart of DO enrichment analysis of the potential targets of flavonoids in citrus plants. **(C)** The bar chart displays the number of citrus plants targets significantly associated with AD pathologies. **(D)** Specific information on the citrus plant targets significantly associated with AD pathologies. **(E)** The upset plot illustrates the overlap among anti-AD targets of citrus plants identified by different strategies. **(F)** Information of the top 20 nodes ranked by degree value in the PPI network constructed from citrus plants targets. **(G)** The bar chart displays the top 30 flavonoids in medicinal and edible citrus plants based on the number of anti-AD targets.

We employed three strategies to identify the anti-AD targets of citrus plants and their corresponding phytochemicals. Firstly, based on the KEGG database, we found that flavonoids in medicinal and edible citrus plants are involved in the Alzheimer disease (hsa05010) through 67 targets (Figure 3A). Secondly, we conducted a DO enrichment analysis to determine the correlation between potential targets of citrus plants and human diseases. A total of 714 DO terms were significantly enriched, with those exhibiting an adjusted *p*-value of less than 0.05 deemed to be significantly enriched. The DO enrichment analysis results showed that the citrus plants targets were significantly enriched in various central nervous system diseases, such as tauopathy (DOID:680), Alzheimer’s disease (DOID:10652), cerebrovascular disease (DOID:6713), dementia (DOID:1307), and Parkinson’s disease (DOID:14330) (Figure 3B). The typical molecular pathological mechanisms of AD involve the amyloid plaques formed by A*β* and tau protein-induced neurofibrillary tangles, whose interactions mediate cognitive dysfunction in AD patients (Nam et al., 2025). Thirdly, our analysis revealed that 24.1% of the citrus plant targets (196 targets) were significantly associated with Aβ and tau pathology (Figure 3C). The potential targets informations of citrus plants significantly correlated with Aβ, tau, Aβ and tau pathologies are presented in Figure 3D. Through the aforementioned three strategies, we have identified a total of 304 anti-AD targets in citrus plants, with overlapping targets among the different strategies (Figure 3E). The 304 anti-AD targets of flavonoids derived from medicinal and edible citrus plants have established a complex PPI network, which consists of 295 nodes and 6,864 edges. Based on their degree values, the top 30 targets, including AKT1, TNF, IL6, TP53, and IL1B, were identified as the core targets for the treatment of AD with medicinal and edible citrus plants (Figure 3F). Our further analysis revealed that the 304 anti-AD targets in medicinal and edible citrus plants correspond to 45 flavonoids. Among these flavonoids, phytochemicals such as quercetin, nobiletin, hesperidin, apigenin, HTMF, tangeretin, hesperetin, diosmetin, gardenin B, kaempferol, isorhamnetin, and naringin possess the highest number of anti-AD targets (Figure 3G).

### 3.4 Key flavonoids of citrus plants that are associated with aβ deposition

**Figure 4:**
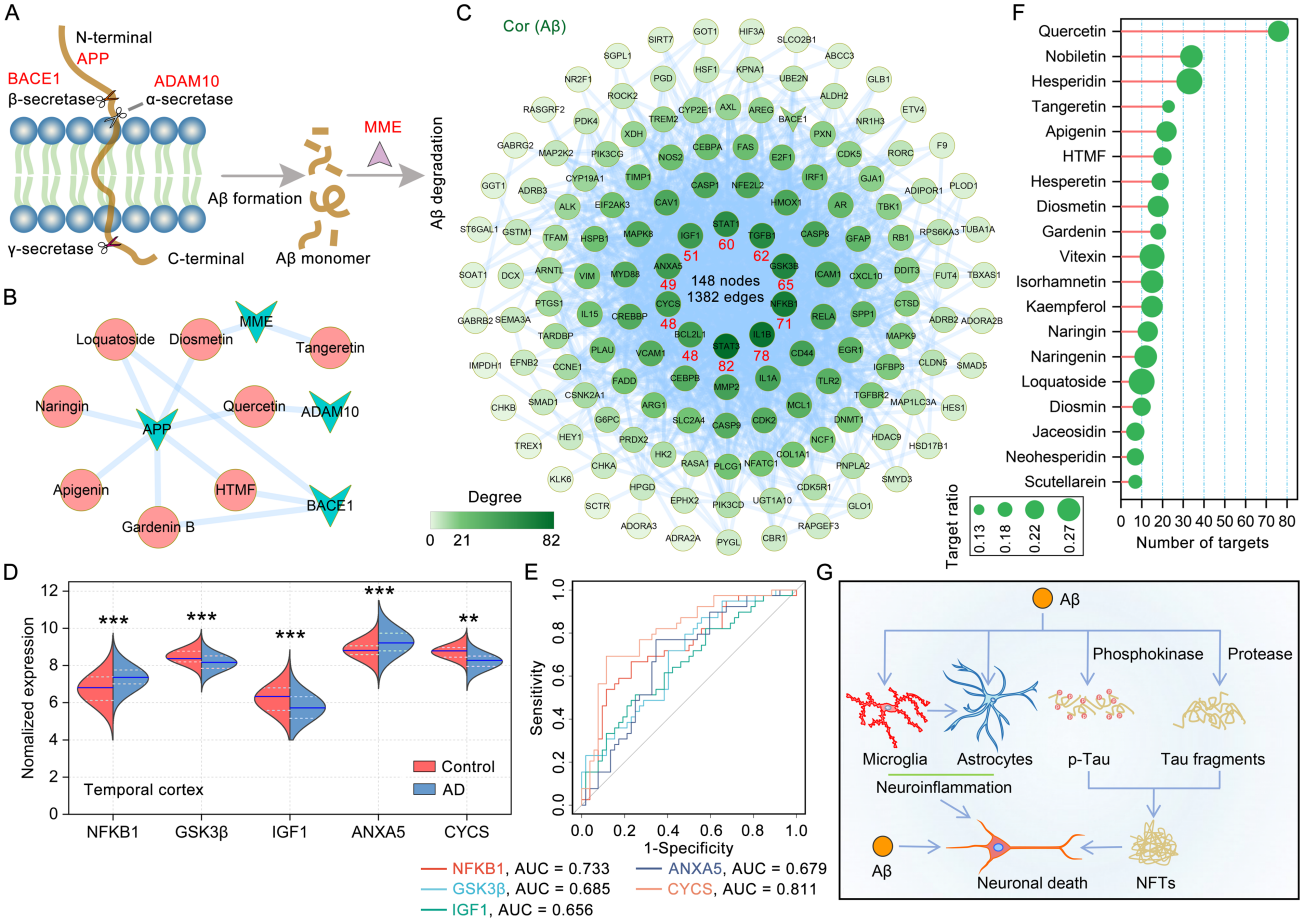
Targets of medicinal and edible citrus plants for Aβ generation and degradation. **(A)** Schematic diagram of the Aβ production and Aβ degradation, with citrus plants targets shown in red font. Aβ is produced by *α*-, β-, and *γ*-secretase-mediated cleavage of APP and efficiently degraded by MME. **(B)** The network of flavonoids-Aβ production and degradation targets. **(C)** PPI network of citrus targets significantly correlated with Aβ pathology. The node color depth is positively correlated with the degree value. **(D)** Split violin plot presented the comparison of expression levels of anti-Aβ targets between the AD and healthy control. Temporal cortex, *n* = 39 in the healthy control group, *n* = 52 in the AD group. **(E)** Multiple ROC curves curves for the diagnostic value of anti-Aβ targets in AD. As a reference, a curve with an AUC of 0.5 was plotted (dashed line). **(F)** Citrus plants flavonoids involved in Aβ pathology, Aβ production and degradation ranked according to the numbers of targets. The target ratio is defined as the proportion of citrus plants anti-Aβ targets to its total targets. **(G)** The mechanisms of Aβ and tau pathology interrelation inducing neuronal death in AD.

Cerebral Aβ deposition represents a significant pathophysiological event in age-related dementia. Aβ exerts neurotoxic effects that compromise the blood–brain barrier (BBB), lead to neuronal and synaptic loss, trigger neuroinflammation, and disrupt synaptic transmission (Kim et al., 2025). Consequently, reducing the Aβ load in the brain has emerged as a crucial strategy for the treatment of AD. APP is a transmembrane protein that is widely distributed in both the brain and peripheral organs, with its primary localization in neurons within the human brain. The production of Aβ depends on the cleavage of APP by *α*-secretase (ADAM10), β-secretases (BACE1) and *γ*-secretases. MME (encodes neprilysin) and IDE (insulin degrading enzyme) are the most prominent Aβ-degrading enzymes (Figure 4A). Based on the four Aβ production- and degradation-related targets of citrus plants shown in Figure 4A, the corresponding flavonoids were identified (Figure 4B). Among these flavonoids, diosmetin acts on both Aβ production and degradation. We further constructed a PPI network of anti-AD targets significantly associated with Aβ pathology in citrus plants. The resulting PPI network contained 148 nodes and 1,382 edges, among which STAT3, IL1B, NFKB1, GSK3B, TGFB1, STAT1, IGF1, ANXA5, CYCS and BCL2L1 have the highest degree values in this PPI network (Figure 4C). Among these targets, NFKB1 and ANXA5 were significantly up-regulated, and Glycogen synthase kinase 3β (GSK3β), IGF1 and CYCS were significantly down-regulated in the temporal cortex in AD patients compared to healthy controls (Figure 4D). ROC curve analysis was used to describe the discrimination accuracy of these targets in the diagnosis of AD. The area under the ROC curve (AUC) is a comprehensive measure of sensitivity and specificity, and the closer it is to 1, the more superior the diagnostic performance of the test. The AUC values, ranked in descending order, are CYCS, NFKB1, GSK3β, ANXA5, IGF1, indicating a sequential decrease in their diagnostic performance for AD (Figure 4E). Among the anti-Aβ flavonoids, quercetin, nobiletin, hesperidin, tangeretin and HTMF were the top six regulators of Aβ generation and degradation (Figure 4F). The deposition of Aβ is the primary pathological hallmark of AD, which can trigger tau pathology, neuroinflammation, synaptic dysfunction, and neuronal death, ultimately leading to dementia (Figure 4G). Thus, the therapeutic targeting of both the production and degradation of Aβ may represent one of the key mechanisms through which citrus plants contribute to the treatment and prevention of AD.

### 3.5 Major effects of the bioactive flavonoids of medicinal and edible citrus plants on tau aggregation

**Figure 5:**
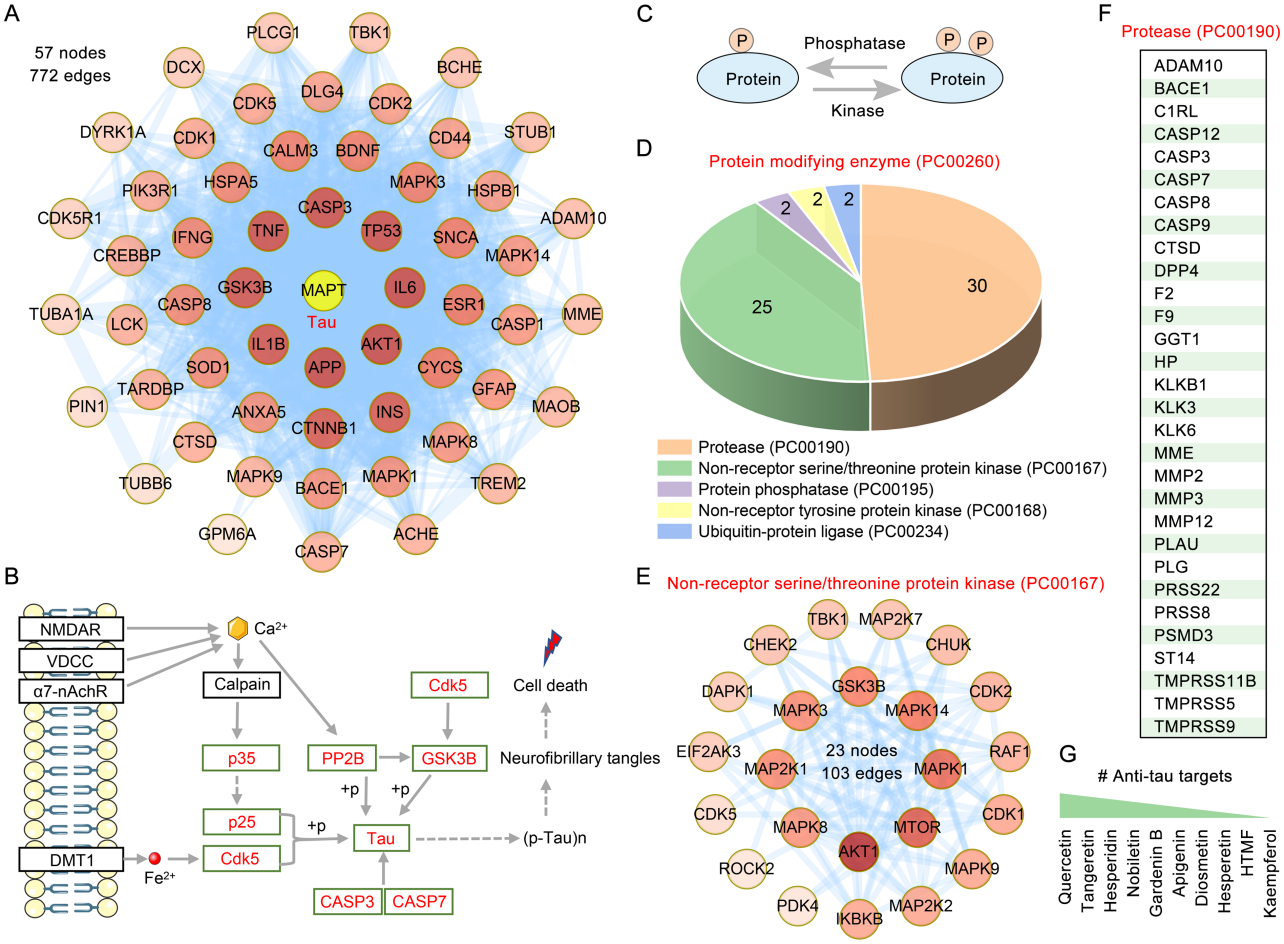
Targets of medicinal and edible citrus plants for tau phosphorylation and degradation. **(A)** The PPI network reveals anti-AD targets in citrus plants that directly interact with tau. **(B)** Schematic diagram of the regulatory mechanism of tau phosphorylation. The red font targets represent the citrus plants targets involved in the regulation of tau phosphorylation. **(C)** The balance between protein kinase and phosphatase activities regulates protein phosphorylation. **(D)** The 3D pie chart displays the citrus plants targets involved in protein modifying enzyme (PC00260). The numbers above each pie chart represent the number of the targets in the given functional class. **(E)** The PPI network of targets involved in the non-receptor serine/threonine protein kinase. Colors of the nodes represent the degree, the darker the color the greater the degree. **(F)** The table presents 30 targets of citrus plants associated with protease (PC00190). **(G)** Citrus plants flavonoids involved in tau phosphorylation and degradation ranked according to the numbers of targets.

The aggregation of tau, such as NTFs, is a significant hallmark of tauopathies. According to the PPI network, among the 304 anti-AD targets of citrus plants, 56 targets directly interact with tau, forming a complex network consisting of 57 nodes and 772 edges (Figure 5A). According to the Alzheimer’s disease pathway (hsa05010), we listed the main AO targets that regulate tau phosphorylation, such as tau, p25, p35, Cdk5, GSK3β, and PP2B (Figure 5B). The phosphorylation status of a protein is the result of the balance between kinase and phosphatase activities (Figure 5C). We further classified the protein functions of the citrus plant targets. The protein modifying enzyme (PC00260) has the highest number of enriched targets, with protein functions such as protease (PC00190), non-receptor serine/threonine protein kinase (PC00167), protein phosphatase (PC00195) and non-receptor tyrosine protein kinase (PC00168) involved (Figure 5D). Protein kinases that belong to the serine/threonine category are enzymes responsible for facilitating the phosphorylation of serine or threonine residues on target proteins. The 25 citrus plants targets are non-receptor serine/threonine protein kinases, which form a complex network consisting of 23 nodes and 103 edges (Figure 5E). Proteases hydrolyze the peptide bonds of proteins into peptides and amino acids, with a total of 30 targets involved in protease (PC00190) in citrus plants (Figure 5F). Among the flavonoids in anti-tau pathology, quercetin, tangeretin, hesperidin, nobiletin and gardenin B were the top five regulators in post-translational modification, or degradation of tau (Figure 5G).

### 3.6 Targets and flavonoids in medicinal and edible citrus plants for regulating neuroinflammation and neuronal death

**Figure 6:**
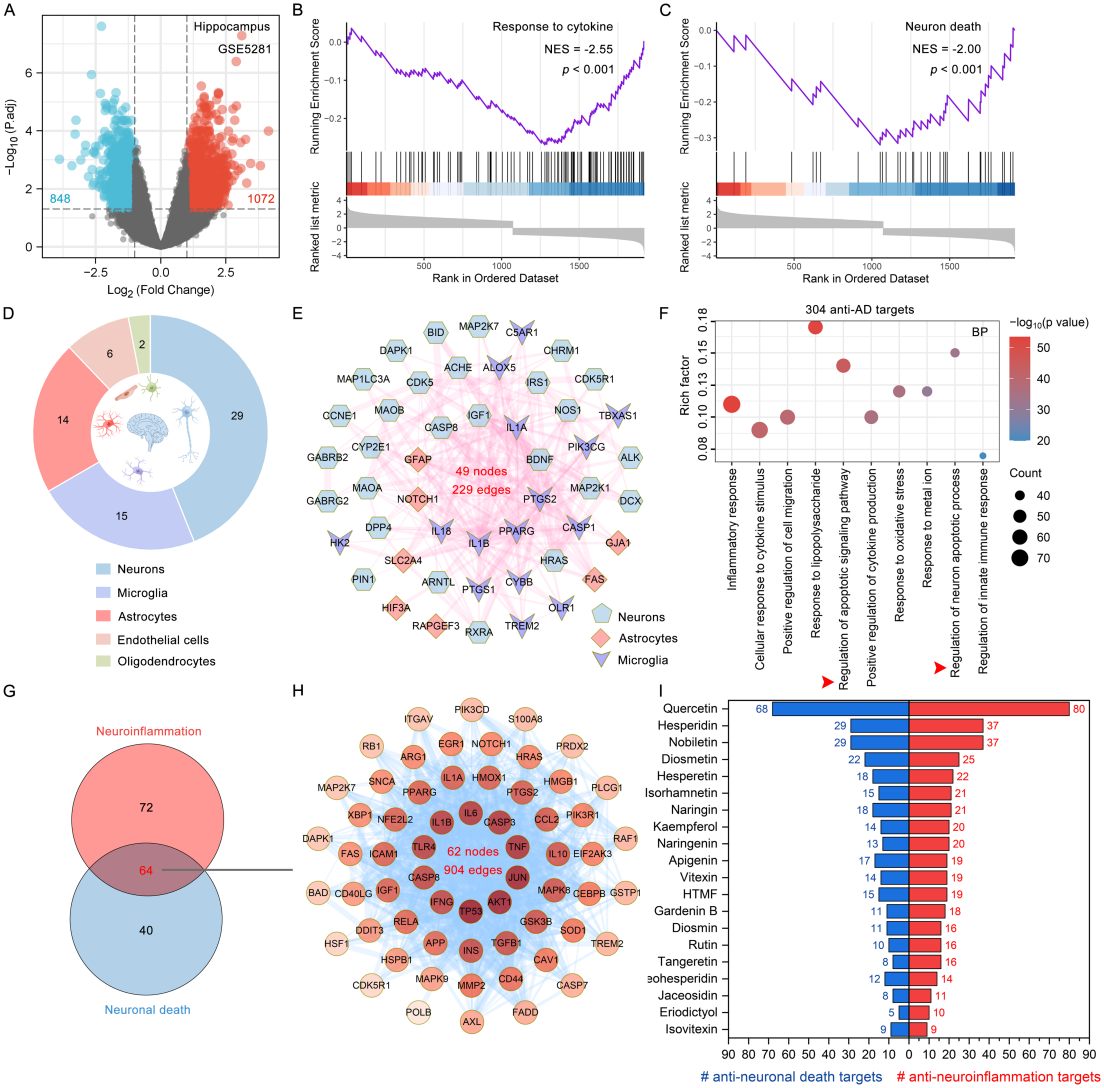
The targets and corresponding flavonoids of citrus plants targeting neuroinflammation and neuronal death. **(A)** Volcano plot of DEGs between the AD and control brain. The threshold parameters used were |Log_2_(fold change)| > 1and *p* adjust < 0.05. **(B,C)** The GSEA enrichment plot revealed that response to cytokine and and neuron death pathways were significantly enriched in the brain tissues of AD patients. **(D)** Anti-AD targets of citrus plants specifically localized to human brain cells such as neurons and microglia. **(E)** The PPI network demonstrates citrus plant targets specifically localized in neurons, microglia, and astrocytes. **(F)** Top 10 significantly enriched biological process terms of citrus plant anti-AD targets are shown as a bubble diagram. X-axis, rich factor (the ratio of targets in the background terms). Bubble size, the number of genes enriched. Bubble color, −Log_10_(*p* value). **(G)** Venn diagram illustrates the common targets enriched between the neuroinflammation and neuronal death. **(H)** The PPI network of common enriched targets between neuroinflammation and neuronal death. **(I)** The top 20 phytochemicals targeting anti-neuroinflammation and neuronal death in citrus plants.

Neuroinflammation is closely related to neuronal apoptosis during the progression of AD (Heneka et al., 2025). The GSE5281 dataset was sourced from the GEO database (Liang et al., 2007). A total of 1,920 DEGs were identified, comprising 1,072 up-regulated and 848 down-regulated genes, after applying the screening criteria of a fold change greater than 2 and an adjusted *p*-value of less than 0.05 (Figure 6A). By utilizing GSEA, we discovered that DEGs are closely associated with both inflammatory responses and neuron death (Figures 6B,C). We further analyzed the brain-specific cellular localization of 304 anti-AD targets in citrus plants, with 29, 15, and 14 targets specifically localized in neurons, microglia, and astrocytes, respectively (Figure 6D). Citrus plant targets specifically localized in neurons, microglia, and astrocytes formed a complex network consisting of 49 nodes and 229 edges based on the STRING database (Figure 6E). The GO enrichment analysis of anti-AD targets in citrus plants also demonstrated that their mechanisms for combating AD are significantly enriched in the biological processes of neuroinflammation and neuronal death (Figure 6F). In the biological processes of neuroinflammation and neuronal death, there are 64 common targets, which together form a complex PPI network consisting of 62 nodes and 904 edges (Figures 6G,H). Among the PPI network, TP53, AKT1, JUN, TNF, CASP3, IL6 and IL1B play crucial roles in the co-regulation of neuroinflammation and neuronal death. The GO cellular component and molecular function categories of 304 anti-AD targets in citrus plants are shown in Supplementary Figure S2. In citrus plants, 44 flavonoids exert brain-protective effects by regulating neuroinflammation and neuronal death, among which quercetin, hesperidin, nobiletin, diosmetin, hesperetin, isorhamnetin and naringin are the most promising regulators of neuroinflammation and neuronal death (Figure 6I).

### 3.7 The interaction between ferroptosis and the classic pathology of AD is an important mechanism for the anti-AD properties of citrus plants

**Figure 7:**
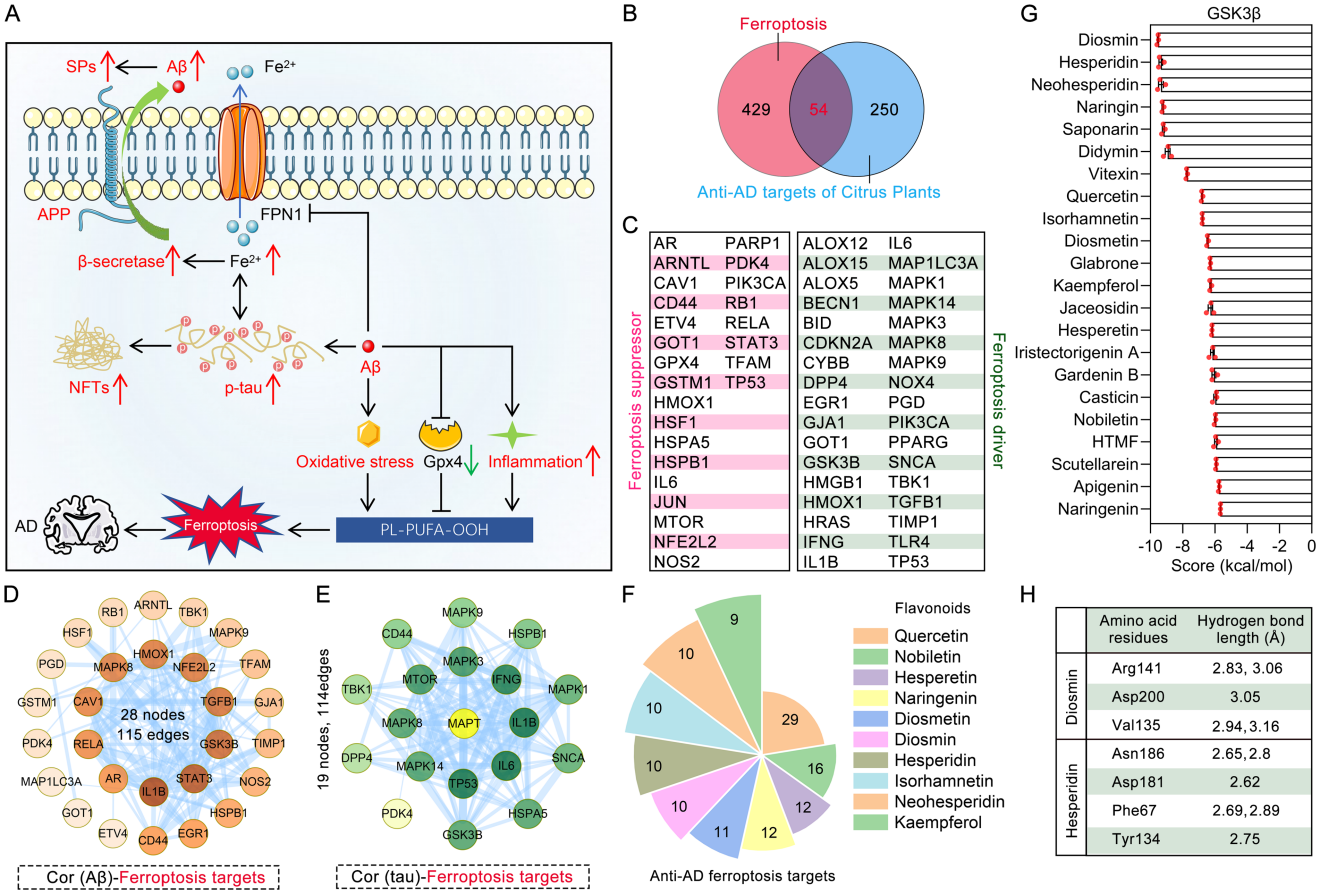
Ferroptosis is one of the mechanisms of flavonoids of citrus plants regulating the pathophysiological processes of AD. **(A)** Schematic illustration of the interaction between ferroptosis and molecular mechanisms of AD pathologies. **(B)** Venn diagram shows the common targets between anti-AD targets in citrus plants and ferroptosis targets. **(C)** Classification of 54 common targets between citrus plants’ anti-AD targets and ferroptosis. Ferroptosis driver, targets that promote ferroptosis; ferroptosis suppressor, targets that prevent ferroptosis. **(D)** PPI network of ferroptosis targets significantly associated with Aβ pathology in citrus plants. **(E)** PPI network of ferroptosis targets significantly associated with tau pathology in citrus plants. **(F)** The primary phytochemicals in citrus plants regulating ferroptosis in AD. **(G)** Bar chart of molecular docking scores between flavonoids of citrus plants and GSK3β (PDB ID: 2O5K) (*n* = 3). **(H)** Amino acid residues involved in the interaction between diosmin, hesperidin and GSK3β and their hydrogen bond lengths.

The pathological mechanism of AD is complex, and ferroptosis has been proven to be closely related to classic AD pathologies such as iron accumulation in the brain, oxidative stress, and neuroinflammation (Figure 7A). Among the 304 anti-AD targets of citrus plants, 54 are ferroptosis targets and are involved in the pathophysiological process of AD (Figure 7B). Specifically, 29 of these targets act as ferroptosis drivers, while 20 function as ferroptosis suppressors (Figure 7C). Among the 54 ferroptosis targets regulated by citrus plants, 28 are implicated in Aβ pathology, resulting in a complex PPI network that consists of 28 nodes and 115 edges (Figure 7D). Moreover, 18 ferroptosis targets are involved in tau (encoded by the MAPT gene) pathology, forming a complex PPI network consisting of 19 nodes and 114 edges (Figure 7E). Among citrus plants, 36 flavonoids have the potential to regulate ferroptosis, with the top 10 phytochemicals named quercetin, nobiletin, hesperetin, naringenin, diosmetin, diosmin, hesperidin, isorhamnetin, neohesperidin and kaempferol having the highest number of ferroptosis regulatory targets. Among citrus plants, 36 flavonoids have the potential to regulate ferroptosis, with the top 10 phytochemicals named quercetin, nobiletin, hesperetin, naringenin, diosmetin, diosmin, hesperidin, isorhamnetin, neohesperidin and kaempferol having the highest number of ferroptosis regulatory targets (Figure 7F). As a key positive regulatory kinase for ferroptosis, GSK3β is associated with both Aβ and tau pathology. This study validated the interactions between 22 flavonoids from citrus plants and GSK3β through molecular docking. The docking scores of all 22 flavonoids with GSK3β were less than −5.5 kcal/mol, among which the molecular docking scores of diosmin, hesperidin, neohesperidin with GSK3β were −9.54, −9.36, −9.34 kcal/mol, respectively (Figure 7G). The crystal structure of GSK3β is a complex with a benzoimidazol inhibitor, named HBM. The molecular docking score of HBM with GSK3β using Ledock software was −8.95 kcal/mol, indicating that diosmin, hesperidin, neohesperidin, naringin, saponarin, and didymin exhibit better binding affinity than the positive small molecule in the same docking system (Supplementary Figure S3). As shown in Figure 7H, diosmin formed hydrogen bonds with multiple amino acids of GSK3β, including Arg141 (hydrogen bond length 2.83, 3.06 Å), Asp200 (hydrogen bond length 3.05 Å), and Val135 (hydrogen bond length 2.94, 3.16 Å). In addition, hesperidin bound with GSK3β by forming 4 hydrogen bonds at Asn186, Asp181, Phe67 and Tyr134 residues. These results indicate that targeting ferroptosis is one of the mechanisms by which citrus plants to ameliorate AD.

### 3.8 Hesperidin protected LPS-induced inflammatory response mediated by the JNK/NFκB signaling pathway

**Figure 8:**
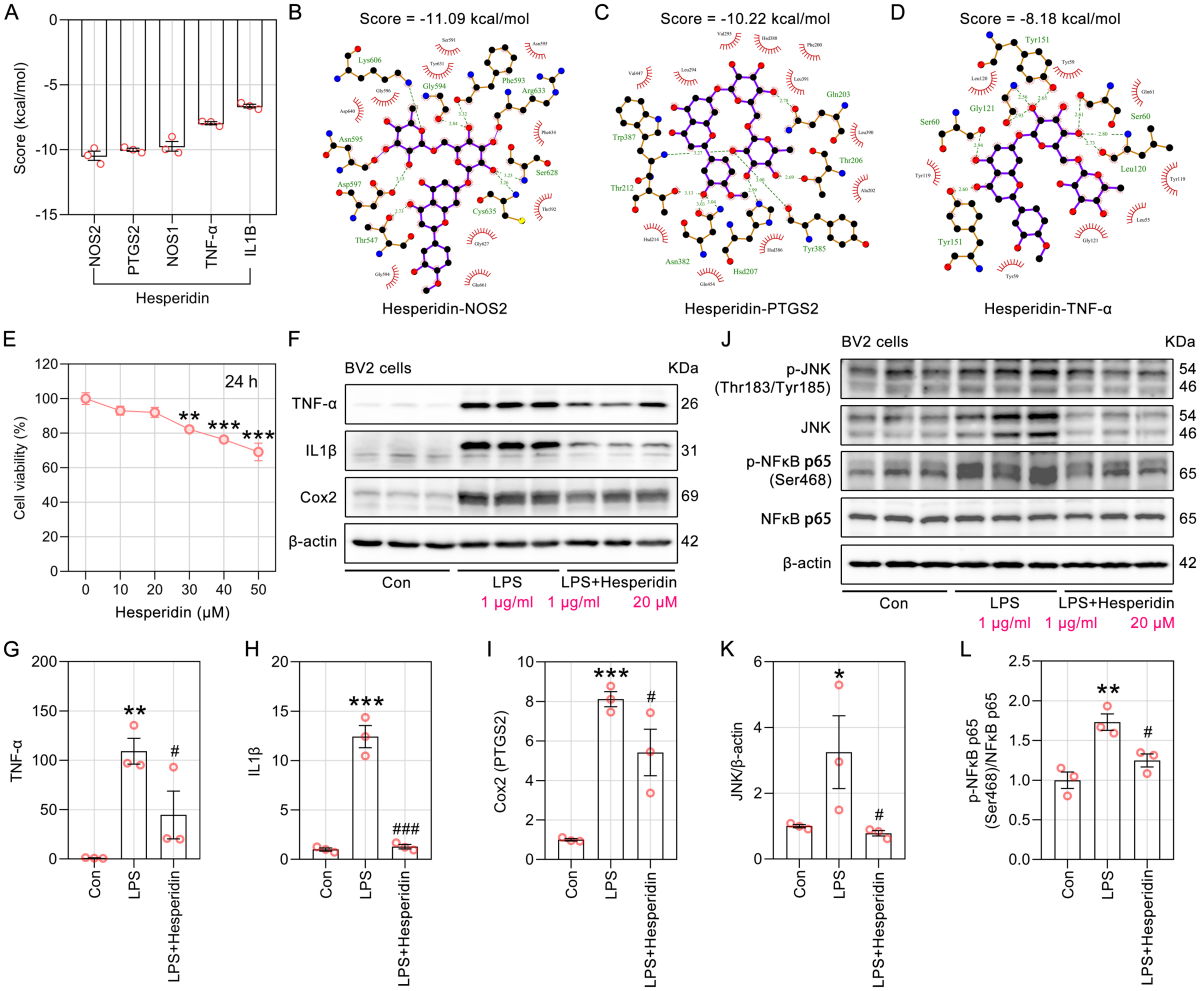
The effect of hesperidin on the levels of pro-inflammatory cytokines in LPS-induced BV2 microglial cells. **(A)** The bar chart displays the molecular docking scores of hesperidin with multiple pro-inflammatory cytokines (*n* = 3). **(B–D)** LigPlus schematic 2D representation of the interactions between hesperidin and NOS2, PTGS2, TNF-α. The amino acid residues of receptor proteins that interact with hesperidin are depicted as brown sticks and labeled in green. **(E)** Viability of BV2 cells treated with hesperidin in graded concentrations (0, 10, 20, 30, 40, 50 μM) for 24 h was conducted by CCK8 assay (*n* = 6). **(F)** Representative immunoblots of TNF-α, IL-1β, and Cox2 proteins in BV2 microglial cells. **(G–I)** Semiquantitative analysis of the relative levels of TNF-α, IL-1β, and Cox2 by densitometric analysis (*n* = 3). **(J)** Hesperidin ameliorates LPS-induced inflammatory responses by inhibiting the JNK/NFκB signaling pathway. **(K,L)** Effects of hesperidin on the p-JNK, JNK, p-NFκB p65 and NFκB p65 levels, as analyzed by quantification of western blotting data (*n* = 3). * *p* < 0.05, **p < 0.01, ***p < 0.001 *vs* Con, # *p* < 0.05, ### *p* < 0.001 *vs* LPS.

Hesperidin is recognized as one of the safest flavonoids derived from citrus plants, and it also exhibits the second highest number of anti-AD targets. The molecular docking results indicate that hesperidin exhibits favorable direct interactions with several common pro-inflammatory cytokines, including TNF-α, IL-1β, and Cox2 (encoded by *PTGS2*) (Figure 8A). Specifically, hesperidin formed 7 hydrogen bonds with 9 amino acid residues in the NOS2 binding pocket (Figure 8B). Hesperidin formed potential interactions with residues Asn382, Gln203, Hsd207, Thr202, Thr212, Tyr385 and Tyr387 of PTGS2 through 8 hydrogen bonds, with an average bond length of 2.99 Å (Figure 8C). Hesperidin forms 8 hydrogen bonds with amino acid residues Gly121, Leu120, Ser60 and Tyr151 in the TNF-α binding pocket, with bond lengths ranging from 2.56 Å to 2.94 Å, indicating strong and stable interactions (Figure 8D). In this study, the molecular docking results serve primarily as structural predictions and preliminary bioinformatics evidence, suggesting that hesperidin has the potential to interact with TNF-α, IL-1β, and Cox2 at the molecular level.

We established an *in vitro* neuroinflammatory model by treating the BV2 microglial cell line with LPS to evaluate the anti-inflammatory effects of hesperidin. The CCK8 results indicated that 20 μM hesperidin for 24 h was the optimal dose for subsequent experiments (Figure 8E). Western blotting analyses revealed that the enhancement of TNF-α, IL-1β, and Cox2 levels by LPS treatment was signcantly attenuated by hesperidin (Figures 8F–I). In addition to direct interactions with pro-inflammatory cytokines, we further investigated the upstream molecular mechanisms by which hesperidin regulates neuroinflammation. JNK is essential for the transcriptional activation and translocation of NFκB, playing a pivotal role in the inflammatory process. Our findings indicate that hesperidin significantly attenuates the LPS-induced elevation in the levels of JNK and its downstream target, p-NFκB p65 (Ser468) (Figures 8J–L). These results suggest that the JNK/NFκB signaling pathway is likely involved in the anti-inflammatory effects of hesperidin.

### 3.9 Naringin protected LPS-induced inflammatory response mediated by the JUN signaling pathway

**Figure 9:**
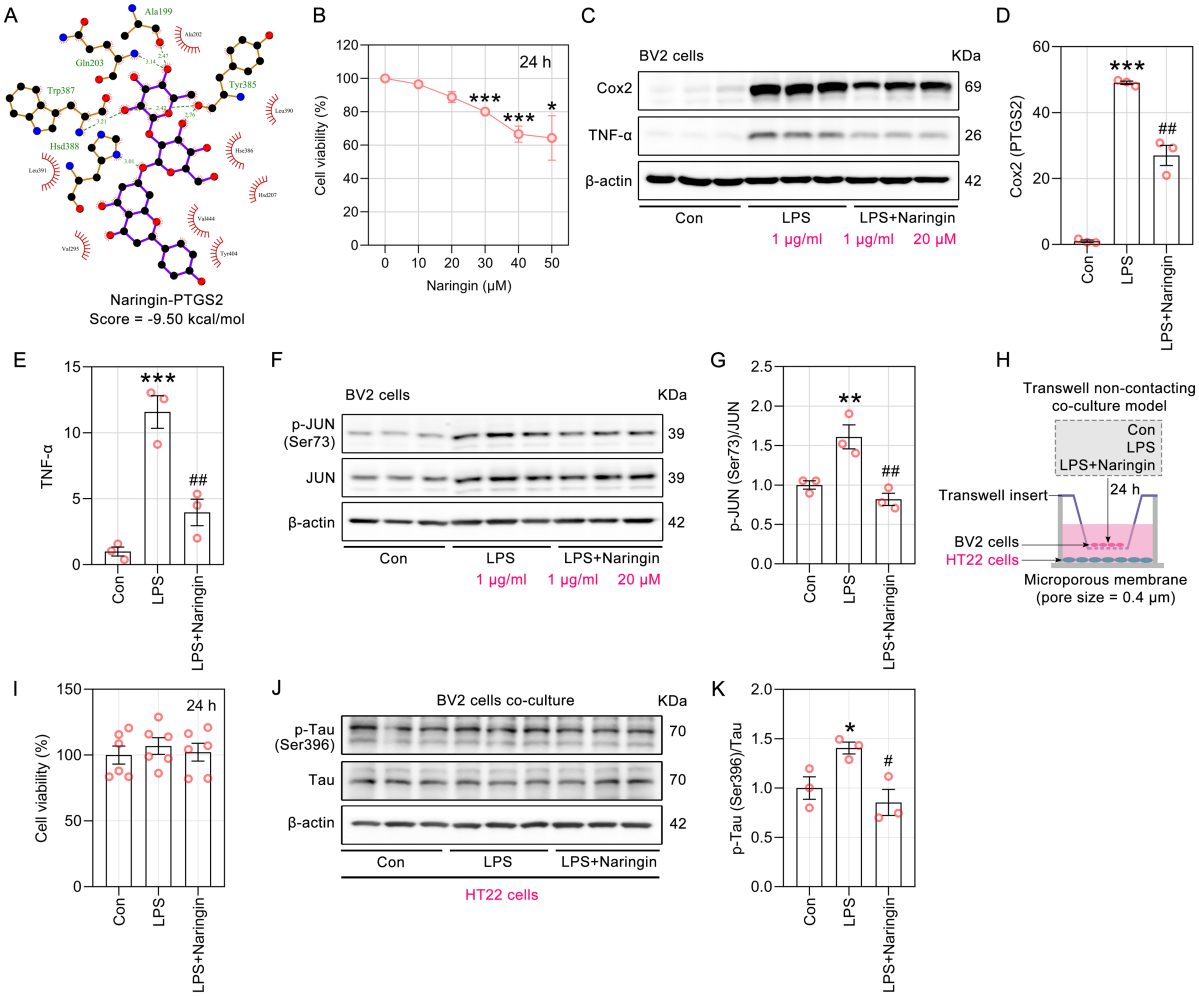
Naringin mitigated LPS-induced pro-inflammatory activation of BV2 cells *in vitro*. **(A)** LigPlus schematic 2D representation of the interactions between naringin and PTGS2. **(B)** Viability of BV2 cells treated with naringin in graded concentrations (0, 10, 20, 30, 40, 50 μM) for 24 h was conducted by CCK8 assay (*n* = 6). **(C)** Representative immunoblots of naringin treatment significantly reduced the levels of Cox2 and TNF-α. **(D,E)** Semiquantitative analysis of the relative levels of Cox2 and TNF-α by densitometric analysis (*n* = 3). **(F)** Representative immunoblots of total JNK, p-JUN(Ser73) and total JUN by Western blotting. **(G)** Ratio of p-JUN to total JUN from immunoblots quantified by densitometry (*n* = 3). **(H)** Schematic diagram for the cell co-culture model of BV2 and HT22 cells. **(I)** After 24 h of co-culture, viability of HT22 cells was determined using CCK8 assay (*n* = 6). **(J)** Western blotting showed the levels of tau phosphorylation at Ser396 and total tau. **(K)** Ratio of p-Tau (Ser396) to total Tau from immunoblots quantified by densitometry (*n* = 3).

Naringin formed six hydrogen bonds with five amino acid residues (Ala199, Gln203, Hsd388, Trp387 and Tyr385) of Cox2 (docking score = −9.50 kcal/mol). The distances between naringin and Ala199, Gln203, Hsd388 and Tyr385 were 2.47, 3.14, 3.21 and 3.01 Å, respectively. Moreover, naringin forms two hydrogen bonds with Trp387 of cox2, with bond lengths of 2.42 and 2.76 Å (Figure 9A). The CCK8 results indicated that 20 μM naringin for 24 h was the optimal dose for subsequent experiments (Figure 9B). To verify the anti-inflammatory effects of naringin, the levels of Cox2 and TNF-α were examined using Western blotting analysis. As shown in Figures 9C–E, there was a significant increase in the levels of Cox2 and TNF-α following LPS treatment. In contrast, treatment with naringin markedly reduced the LPS-induced elevation of Cox2 and TNF-α (*p* < 0.01 compared to the LPS group). The Western blotting results indicated that exposure to LPS led to an increase in the levels of p-JUN(Ser73), while treatment with naringin significantly reversed the aforementioned changes (Figures 9F,G). These results suggest that naringin exerts anti-inflammatory effects by inhibiting JUN signaling pathway. Subsequently, a co-culture system was used to assess the effects of activated microglial cells BV2 on the neuronal cell line HT22 (Figure 9H). After 24 h of co-culture, no significant differences in cell viability were observed among the three groups of HT22 cells (Figure 9I). We confirmed that activated microglia significantly increased the phosphorylation of Tau at the Ser422 site in HT22 cells. Furthermore, treatment with naringin in BV2 cells was found to ameliorate the abnormal elevation of Tau phosphorylation (Figures 9J,K).

## 4 Discussion

**Figure 10:**
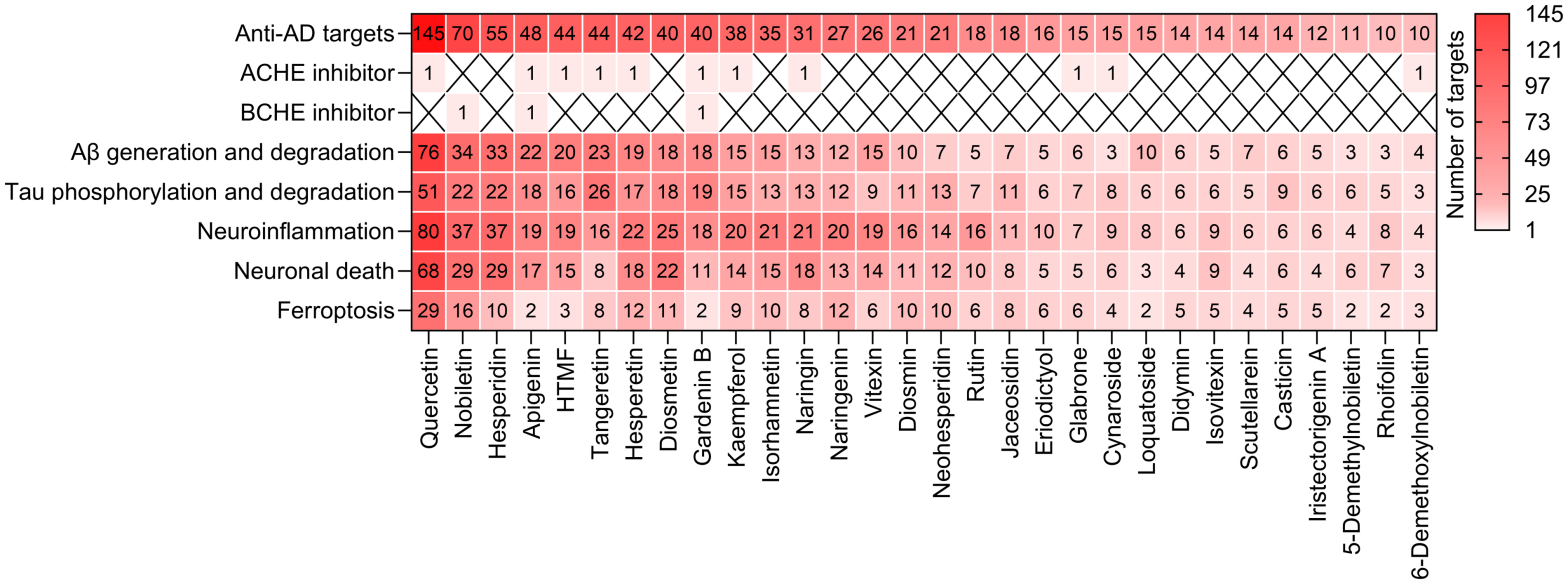
The heatmap summarizes the number of targets for citrus plant phytochemicals that regulate various pathological processes associated with AD. The letter “X” indicates that the corresponding data is not applicable.

Medicinal and edible citrus plants present numerous advantages in the treatment of AD or neurodegenerative dementia. Firstly, their safety profile is exceptional, rendering them particularly suitable for the long-term intervention requirements associated with AD. The course of AD spans several years to over a decade, requiring long-term continuous intervention, with safety being the core prerequisite for prolonged treatment. Citrus plants, as widely consumed fruits globally, have been validated through thousands of years of dietary practice to have no significant toxic or side effects. This study primarily focuses on the natural small molecule flavonoids found in six medicinal and edible citrus plants, which have clear human metabolic pathways and are not prone to accumulation in the body. Secondly, flavonoids in medicinal and edible citrus plants exhibit diverse mechanisms of action, simultaneously intervening in multiple pathological pathways of AD, holding the potential to delay or even halt the progression of AD at its source. Based on the results obtained, we summarize the number of targets of citrus plant phytochemicals that regulate anti-cholinesterase activity, Aβ generation and degradation, tau phosphorylation and degradation, neuroinflammation, neuronal death, and ferroptosis (Figure 10). Thirdly, the resources of medicinal and edible citrus plants are abundant and easily accessible, presenting significant potential for industrialization. Fourthly, medicinal and edible citrus plants exhibit both preventive and therapeutic properties, making them valuable throughout the entire continuum of AD prevention and treatment. Fifthly, medicinal and edible citrus plants, which serve a dual purpose as both medicine and food, thus significantly enhance patient treatment compliance.

This study focuses on six medicinal and edible citrus plants, all of which are rich in flavonoids. The total flavonoid content, ranked from highest to lowest, is as follows: *Fructus Aurantii Immaturus*, *Exocarpium Citri Grandis*, *Fructus Aurantii*, *Zhique*, *Citrus Reticulata Blanco*, and *Citri Sarcodactylis Fructus* (He et al., 2020). Flavanones and polymethoxylated flavones are the two major classes of flavonoids found in medicinal and edible citrus plants. Flavanones mainly include naringin, narirutin, naringenin, hesperidin, neohesperidin, hesperetin, and eriocitrin. Notably, there are significant variations in the types and concentrations of flavanones present among different citrus plants. The primary flavanones identified in *Zhique* are naringin and hesperidin, with respective concentrations of (79.59 ± 0.21) mg/g and (3.35 ± 0.20) mg/g (Cheng et al., 2017). The main flavanones identified in *Fructus Aurantii* and *Fructus Aurantii Immaturus* are naringin, hesperidin, and neohesperidin. The contents of naringin, hesperidin, and neohesperidin in *Fructus Aurantii* were 544.22, 8.44, and 239.59 mg/kg, respectively, while in *Fructus Aurantii Immaturus*, the contents were 676.23, 113.05, and 431.16 mg/kg, respectively. The content of flavanones in *Fructus Aurantii Immaturus* was generally higher than that in *Fructus Aurantii* (Li et al., 2019). *Citrus Reticulata Blanco* mainly contains hesperidin, accounting for approximately 50.2% to 67.6% of the total flavonoid content. The total flavonoid content of *Citrus Reticulata* Blanco at different aging times ranges from (102.50 ± 3.94) to (164.04 ± 8.16) mg/g (Yu et al., 2022).

AChE is a well-established target for the symptomatic treatment of AD, effectively alleviating symptoms and enhancing cognitive and functional impairments. Its overactivation results in the substantial degradation of the neurotransmitter ACh within the brain, which subsequently leads to synaptic transmission disorders and cognitive decline. Although the commonly used AChE inhibitors in clinical practice can alleviate symptoms, they are associated with significant side effects and exhibit a singular mode of action. In medicinal and edible citrus plants, 16 flavonoids have been identified to target AChE. The molecular docking results showed that 12 flavonoids exhibited a high affinity for binding to AChE, showing superior docking scores when compared to five commonly used clinical AChE inhibitors. Furthermore, subsequent MD simulations confirmed that the kaempferol-AChE complex maintained excellent conformational stability throughout the simulation period, as indicated by stable RMSD values. The present in silico analysis offers a solid theoretical foundation for subsequent drug development, however, definitive conclusions must be supported by rigorous experimental evidence.

Flavonoids found in medicinal and edible citrus plants can simultaneously inhibit AChE and ameliorate other pathological aspects of AD, thereby creating a synergistic effect through multiple active phytochemicals (Hussain et al., 2025). As a 3-O-glycoside derivative of quercetin, isoquercitrin exhibits the optimal interaction affinity with AChE. In addition, isoquercitrin also exhibits potent anti-inflammatory, anti-oxidant, and anti-tumor properties (Owczarek-Januszkiewicz et al., 2022). Consistent with the findings of this study, isoquercitrin treatment has also been demonstrated to significantly alleviate neuroinflammation in AD. In LPS-induced microglia, RAW 264.7 macrophages, and mouse hippocampus, treatment with isoquercitrin effectively reduced the production of NO, lipid peroxidation, and the levels of iNOS, ROS, Cox2, as well as decreased the expression of multiple pro-inflammatory cytokines (TNF-α, IL1β, IL-6) (Kim et al., 2022; Lee et al., 2022).

The pathophysiological essence of AD is characterized by a network imbalance that arises from the interaction of multiple pathological processes, rather than being attributed to a single target abnormality. In the pathogenesis of AD, key pathological processes such as Aβ deposition, tau hyperphosphorylation, neuroinflammation, neuronal death, and ferroptosis are intricately intertwined, creating a complex network of pathological vicious cycles (Long et al., 2022a,b; Wang et al., 2023). Ferroptosis is a new form of programmed cell death that is distinct from other modalities of cell death, such as apoptosis and necrosis. Iron metabolism disorders leading to iron overload affect the β-amyloid precursor protein (APP), increasing Aβ production while inhibiting its normal metabolism. This process accelerates the misfolding of Aβ and the aggregation of amyloid plaques (Derry et al., 2020; Gong et al., 2019). Meanwhile, iron metabolism disorders lead to iron deposition in the brain, which induces phosphorylation of tau protein and subsequently promotes the formation of neurofibrillary tangles (Nikseresht et al., 2019; Yan and Zhang, 2020). In models of neurodegenerative diseases induced by tau protein, significant abnormalities in iron metabolism and evidence of iron overload are consistently observed. After reducing intracellular iron levels in neurons using the iron chelator deferiprone, there was a significant decrease in abnormal Aβ deposition and Tau hyperphosphorylation associated with iron overload. Additionally, this treatment reduced ferroptosis in the cortical and hippocampal neurons of dementia animal models and alleviated cognitive impairment (Plascencia-Villa and Perry, 2021).

Flavonoids exhibit strong antioxidant activity and neuroprotective properties (Shen et al., 2022). Flavonoids effectively inhibit ferroptosis by activating the Nrf2 signaling pathway, which leads to the upregulation of GPX4 and HO-1 expression while simultaneously downregulating ACSL4 expression (Živanović et al., 2024). In this study, 36 flavonoids derived from citrus plants targeted 54 ferroptosis-related targets to exert anti-AD effects. GSK3β is a major kinase closely associated with the hyperphosphorylation of tau in AD. GSK3 regulates over 40 putative phosphorylation sites on tau, of which at least 29 are hyperphosphorylated in the brains of individuals with AD (Hanger et al., 2009). In addition, GSK3β also promotes the activity of β- and *γ*-secretases, leading to the accumulation of Aβ peptides and thereby facilitating the formation of Aβ plaques. GSK3β can also enhance the production of inflammatory factors such as IL-1β, IL-6, and TNFα, thereby exacerbating neuroinflammation in AD. Furthermore, abnormal GSK3β activity negatively impacts neurite outgrowth and the regulation of synaptic plasticity, ultimately leading to neuronal death (Ning et al., 2025). GSK3β serves as a positive regulator of ferroptosis, therefore, both selective inhibition of GSK3β and its genetic knockout significantly enhance cellular resistance to ferroptosis (Wang L. et al., 2021).

Multiple studies have confirmed that GSK3β is the key molecule linking ferroptosis to A*β* deposition (Gui et al., 2024; Karati et al., 2025; Zhang et al., 2022). In the 3 × Tg AD mouse model, Schisandrin B inhibits GSK3β activity, thereby regulating the Nrf2/GPX4 pathway, suppressing neuronal ferroptosis, and improving cognitive and neuropathological impairments (Ding et al., 2025). GSK3β plays a critical role in ambient particulate matter-induced ferroptosis of hippocampal cells, and the GSK3β inhibitor (LY2090314) can block the Nrf2/GPX4 pathway to inhibit this process (Gui et al., 2024). Inhibition of ferroptosis directly obstructs the pathological progression associated with Aβ. Differential gene expression analysis of RNA sequencing from AD organoids revealed the activation of the ferroptosis pathway. The ferroptosis inhibitor ferrostatin-1 effectively suppressed the formation of Aβ-like aggregates in cerebral organoids derived from human AD iPSCs, restored ferritin levels, and reduced lipid peroxidation (Majerníková et al., 2024). In addition, ferroptosis leads to abnormal aggregation of tau, and the ferroptosis inhibitor ferrostatin-1 effectively alleviates tau aggregation, with its mechanism being associated with GSK3β (Wang S. et al., 2022). A total of 22 flavonoids in citrus plants exert anti-AD effects by targeting GSK3β, including quercetin, nobiletin, naringin, hesperidin, hesperetin, etc. Through molecular docking system calculations, this study confirmed the binding interactions of 22 flavonoids with human GSK3β. The molecular docking results indicated that naringin binds to the ATP pocket of GSK3β, forming stable hydrogen bonds with Lys85, Asn95, Lys183, and Ser203 (Mokhtar et al., 2024). In summary, inhibiting GSK3β simultaneously blocks both ferroptosis and the pathological progression of AD, thereby providing a critical intervention target for AD. Further experimental validation is necessary to ascertain whether citrus flavonoids inhibit GSK3β, thereby blocking ferroptosis and mitigating the pathological progression of AD. This highlights the synergistic advantages of multi-component and multi-target effects in both medicinal and edible citrus plants.

Despite it has many strengths, this study has some limitations. Firstly, although many bioactive components of citrus plants have shown efficacy in preclinical studies, only a few medicinal herbs and their active constituents have undergone clinical trials. Subsequent studies should conduct large-scale, long-term follow-up randomized controlled clinical trials. Secondly, the bioactive phytochemicals in citrus plants, such as naringenin and nobiletin, are primarily concentrated in the fruit peel and pulp fibers, with their natural content generally below 1%. Therefore, daily consumption of citrus fruits may not effectively intervene in AD or achieve the required therapeutic concentration. Thirdly, flavonoids found in medicinal and edible citrus plants should be seen as additional or preventive measures and not as substitute for current clinical treatment for AD. Additionally, not all flavonoids have undergone validation in animal or cellular models related to AD. Thus, these compounds need to be confirmed through different AD animal models and clinical studies prior to their use in patient treatment.

## 5 Conclusion

This study comprehensively investigated the flavonoids in medicinal and edible citrus plants targeting multiple pathological aspects of AD and their mechanisms of action, based on network pharmacology, molecular docking, and *in vitro* validation. The study identified 45 flavonoids in medicinal and edible citrus plants that correspond to 304 AD-related targets, which are involved in multiple pathophysiological processes. Quercetin, nobiletin, hesperidin, apigenin, HTMF, tangeretin, hesperetin, diosmetin, gardenin B, kaempferol, isorhamnetin and naringin have been identified as the key flavonoids of citrus plants that regulate the pathogenesis of AD in a multitargeted manner. The flavonoids of citrus plants primarily regulate the core targets AKT1, TNF, IL6, TP53, IL1B, STAT3, INS, JUN, CASP3 and CTNNB1. Our findings indicate that targeting ferroptosis is one of the mechanisms by which citrus plants to ameliorate AD. Quercetin, nobiletin, hesperetin, naringenin and diosmetin have been identified as potential potential regulators of ferroptosis, as they interact with a broader range of ferroptosis-related targets. The various citrus plants flavonoids examined in this study exhibit significant potential for clinical translation, particularly in the early prevention and adjuvant treatment of AD. Furthermore, they offer a novel approach for the high-value utilization of medicinal and edible resources derived from citrus plants.

## Data Availability

The original contributions presented in the study are included in the article/Supplementary material, further inquiries can be directed to the corresponding author.

## Footnotes

1
http://www.swissadme.ch/index.php

2
https://tox.charite.de/protox3/

3
http://47.92.70.12/

4
http://pantherdb.org/

5
http://www.alzdata.org/

6
http://www.bioinformatics.com.cn/

7
http://www.lephar.com/software.htm

8
https://www.ebi.ac.uk/thornton-srv/software/LigPlus/

9
https://string-db.org

## Supplementary material

SUPPLEMENTARY FIGURE S1 The distribution of flavonoids in six medicinal and edible citrus plants. The red diamond indicates that the flavonoid has been identified in the corresponding citrus plant, while the green circle indicates that the flavonoid has not been identified in the corresponding citrus plant.

SUPPLEMENTARY FIGURE S2 GO cellular component **(A)** and molecular function **(B)** categories of 304 anti-AD targets in citrus plants.

SUPPLEMENTARY FIGURE S3 Molecular docking of a benzoimidazol inhibitor HBM with GSK3β. **(A)** Molecular docking score and optimal docking pose between HBM and GSK3β. **(B)** LigPlus schematic 2D representation of HBM-GSK3β interactions.

## Glossary

Aβ: β-amyloid
ACh: Acetylcholine
AChE: Acetylcholinesterase
AD: Alzheimer’s disease
APP: β-amyloid precursor protein
AUC: Area under the ROC curve
BBB: Blood–brain barrier
BChE: Butyrylcholinesterase
CCK8: Cell counting kit-8
Cox2: Cyclooxygenase 2
DEGs: Differentially expressed genes
DO: Disease ontology
FDA: Food and drug administration
GAFF2: General Amber Force Field
GO: Gene ontology
GSK3β: Glycogen synthase kinase 3β
GSEA: Gene set enrichment analysis
HTMF: 5-Hydroxy-3′,4′,6,7-Tetramethoxyflavone
MD: Molecular dynamics
NES: Normalized enrichment score
PPI: Protein–protein interaction
RMSD: Root mean square deviation
RO5: Lipinski’s rule of five
ROC: Receiver operating characteristic
SEM: Standard error of mean
