# Supplementary figures and images for: Integrating network pharmacology and experimental validation strategies to investigate the mechanisms and key flavonoids in medicinal and edible citrus plants against Alzheimer’s disease
Source: Front Aging Neurosci. 2026 Apr 28;18:1801263. doi: 10.3389/fnagi.2026.1801263 (PMC13160911; doi:10.3389/fnagi.2026.1801263)

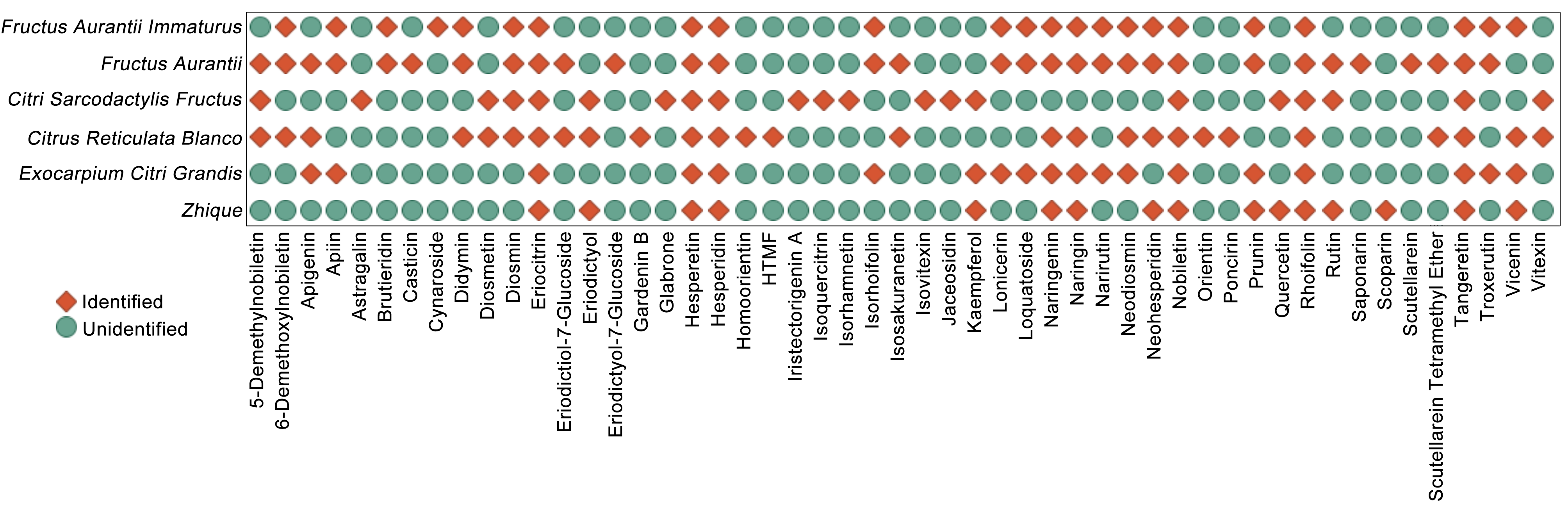

Supplement: SUPPLEMENTARY FIGURE S1 — The distribution of flavonoids in six medicinal and edible citrus plants. The red diamond indicates that the flavonoid has been identified in the corresponding citrus plant, while the green circle indicates that the flavonoid has not been identified in the corresponding citrus plant. [file Image_1.tif]

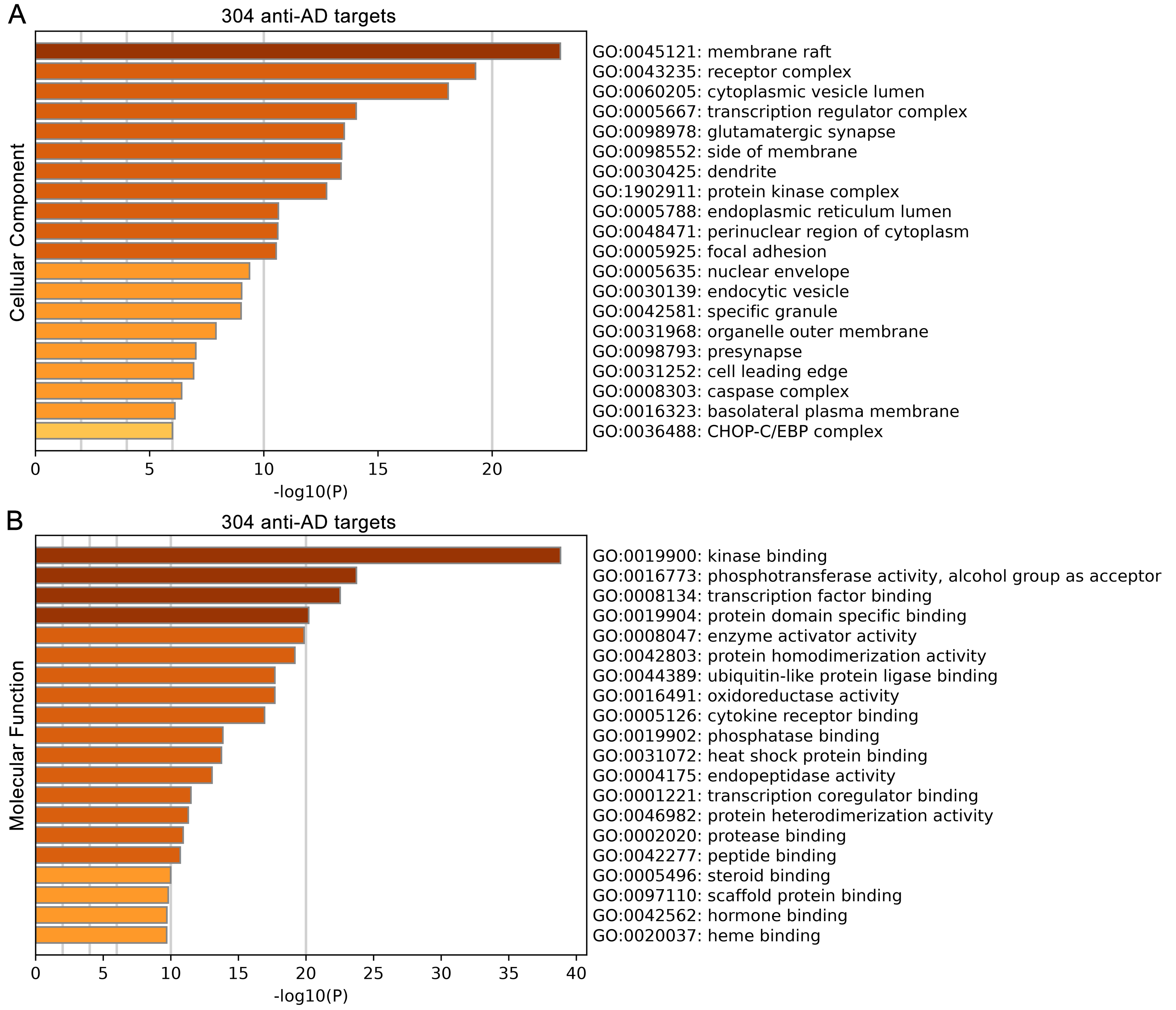

Supplement: SUPPLEMENTARY FIGURE S2 — GO cellular component (A) and molecular function (B) categories of 304 anti-AD targets in citrus plants. [file Image_2.tif]

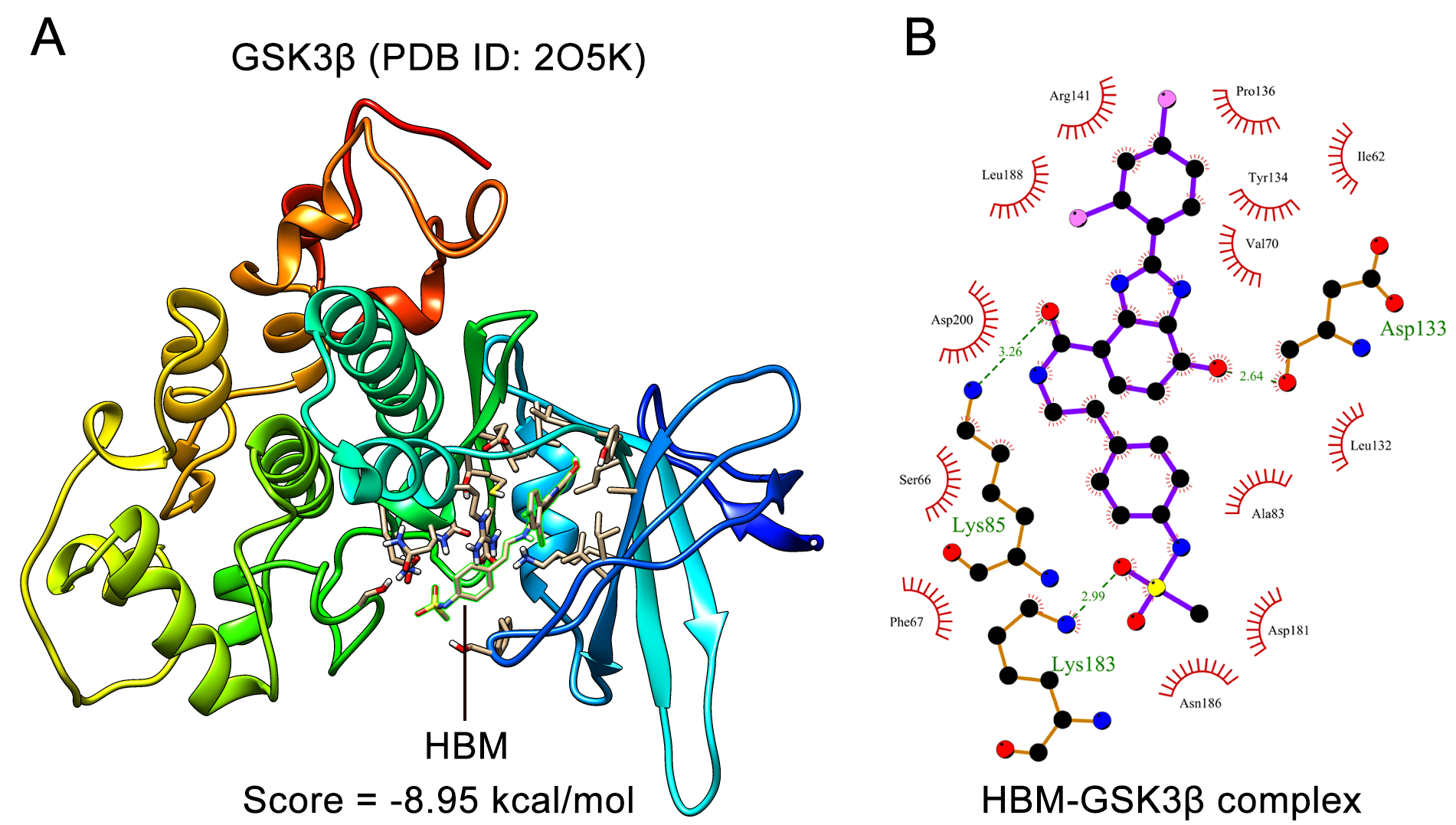

Supplement: SUPPLEMENTARY FIGURE S3 — Molecular docking of a benzoimidazol inhibitor HBM with GSK3β. (A) Molecular docking score and optimal docking pose between HBM and GSK3β. (B) LigPlus schematic 2D representation of HBM-GSK3β interactions. [file Image_3.tif]
